# Supplementary material for: Detection of QTLs for Plant Height Architecture Traits in Rice (Oryza sativa L.) by Association Mapping and the RSTEP-LRT Method
Source: Plants (Basel). 2022 Apr 6;11(7):999. doi: 10.3390/plants11070999 (PMC9002822; doi:10.3390/plants11070999)
Supplement: Supplementary file 1 [file plants-11-00999-s001.zip › plants-1597885-supplementary.pdf]

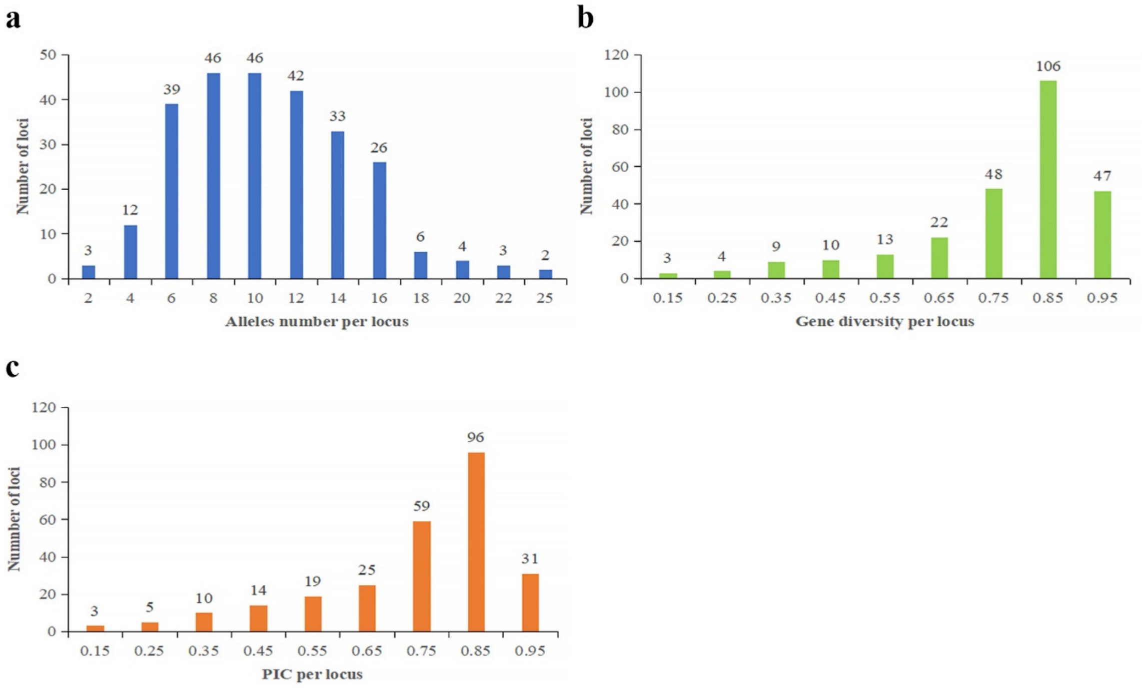

Figure S1. Distribution of genetic diversity of 262 SSR loci across 504 rice accessions. (**a**): Allele number per locus; (**b**): Genetic diversity per locus; (**c**): PIC per locus.

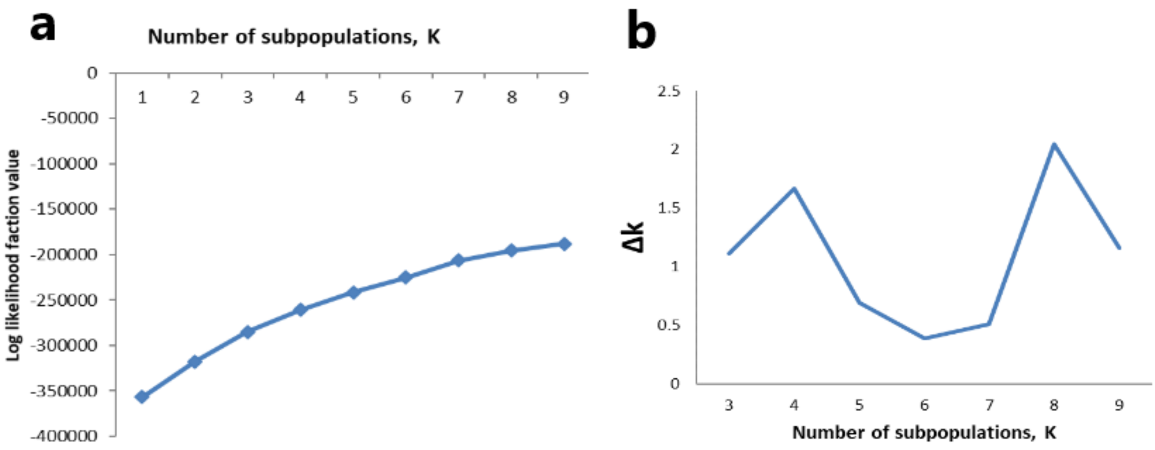

Figure S2. Changes in the log-likelihood function value (**a**), and in ΔK values (**b**) with the number of subpopulations.

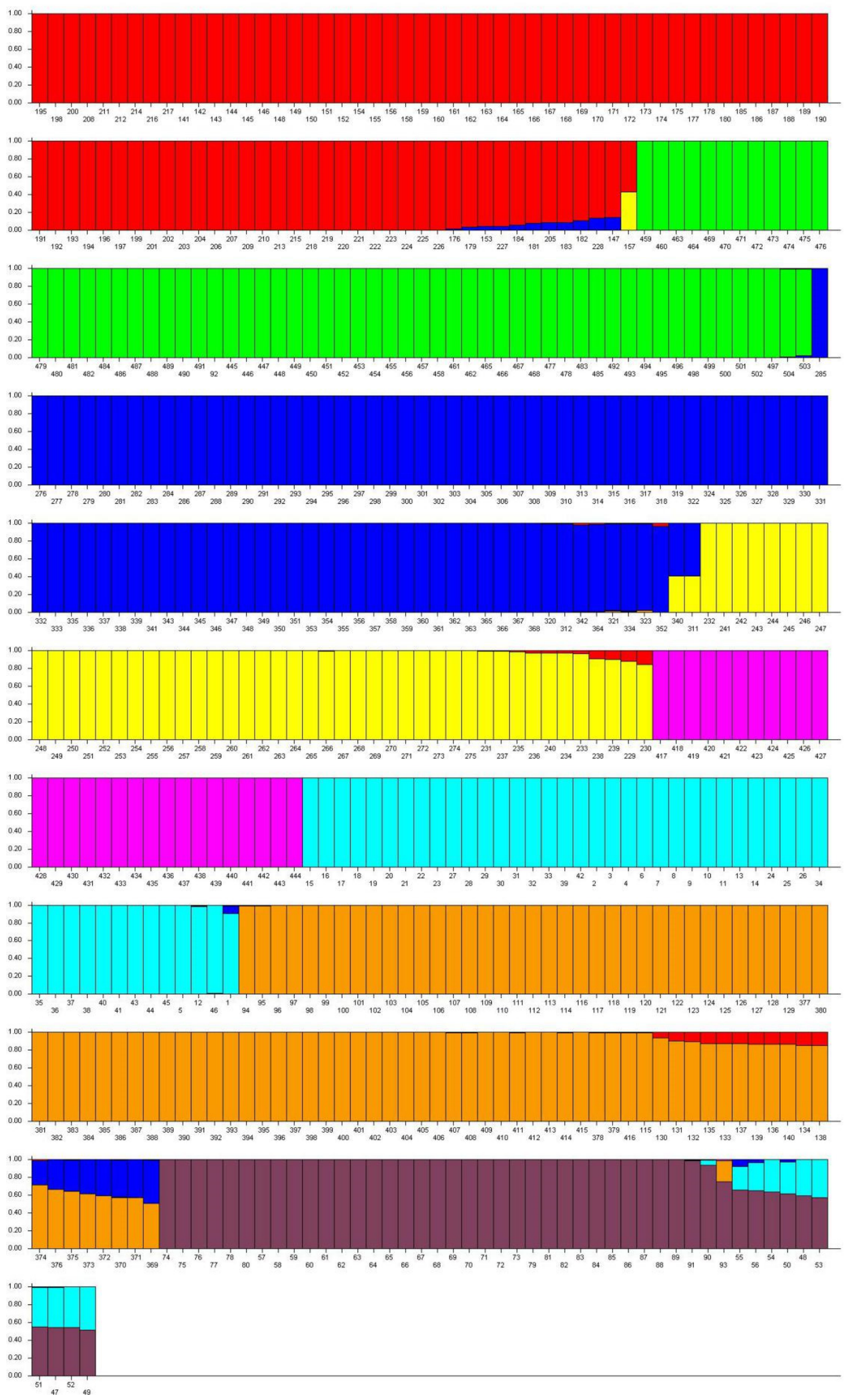

Figure S3. 504 rice variety belonging to eight subpopulations, calculated by STRUCTURE software. Each vertical bar represents an accession and within each vertical bar, the colored subsections represent membership coefficient (Q) of

the accession to different clusters. Identified subpopulations are Sub-pop 1 (red color), Sub-pop 2 (green color), Sub-pop 3 (navy blue color), Sub-pop 4 (yellow color), Sub-pop 5 (pink color), Sub-pop 6 (light blue color), Sub-pop 7 (brown color) and Sub-pop 8 (purple color). The numbers in the X-axis stand for variety code corresponding to Table S1.

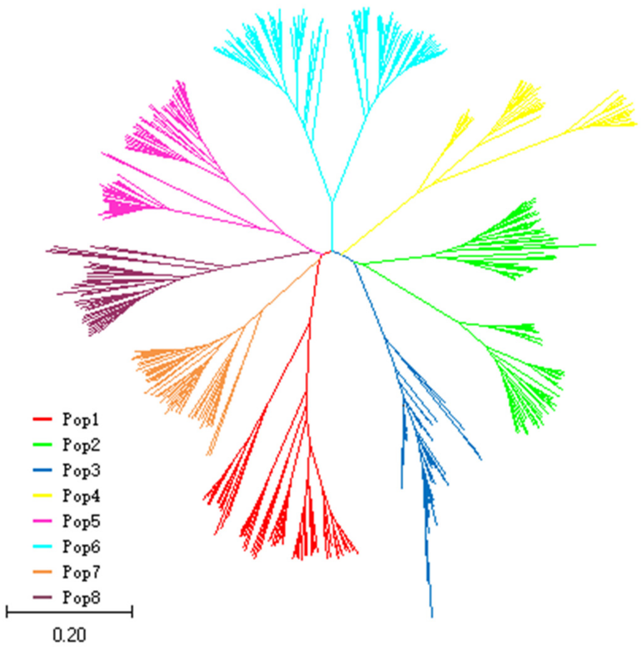

Figure S4. Neighbor joining tree for the 504 accessions constructed using Nei's (1983) genetic distance.

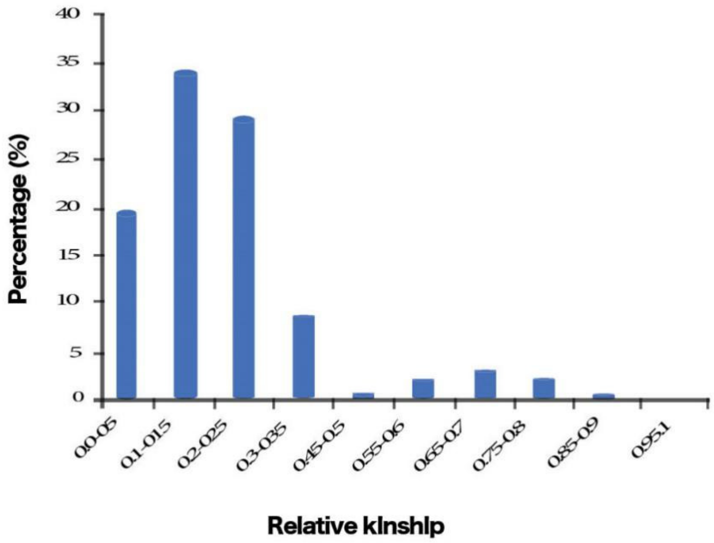

Figure S5. Distribution of pair-wise kinship coefficients among 474 rice accessions based on 262 SSR markers.

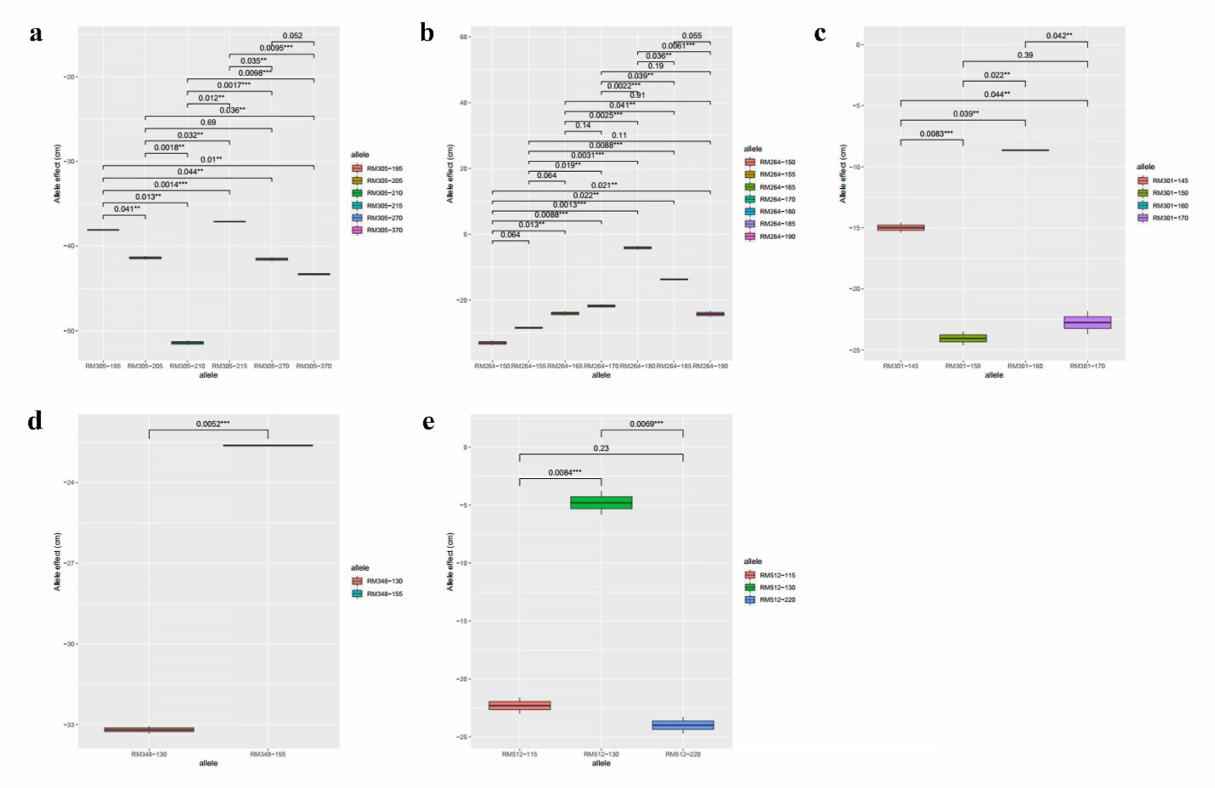

Figure S6. Pairwise *t* test for plant height. ** and *** denote significant at *P*<0.05 and *P*<0.01, respectively.

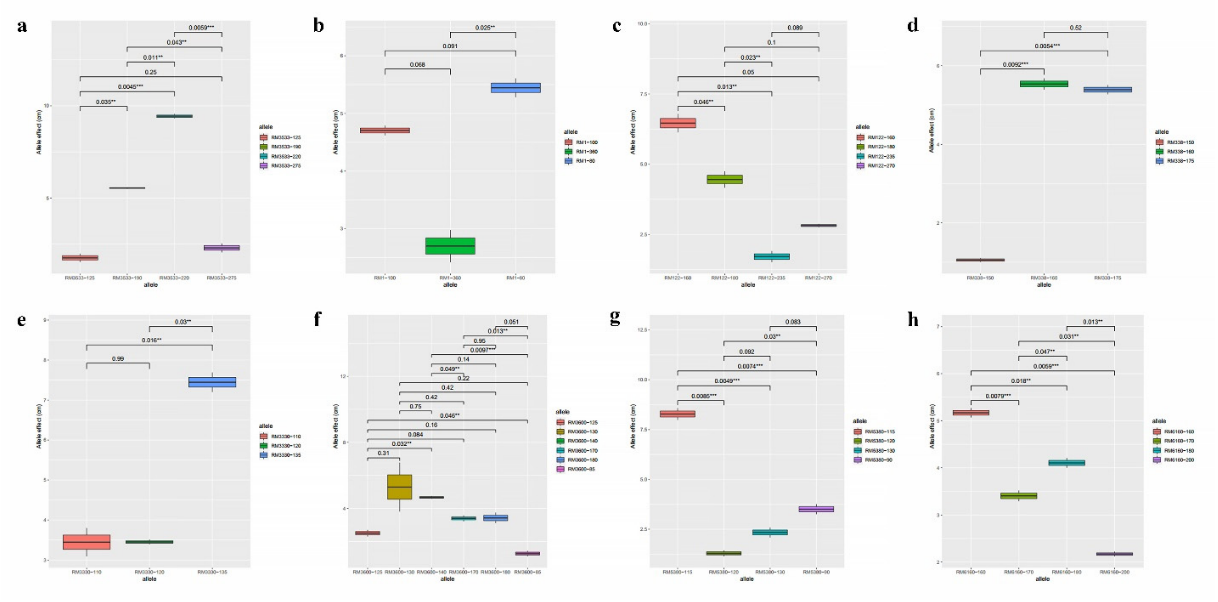

Figure S7. Pairwise *t* test for panicle length. ** and *** denote significant at *P*<0.05 and *P*<0.01, respectively.

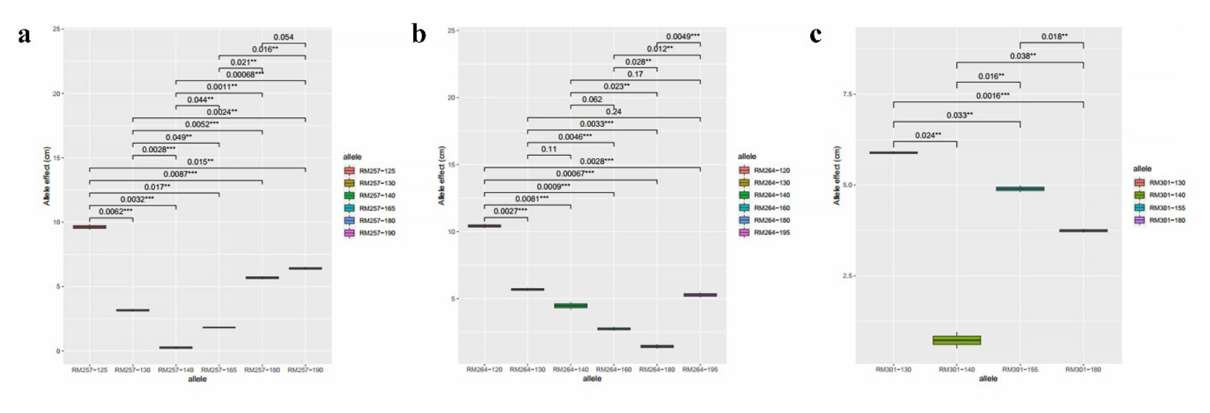

Figure S8. Pairwise *t* test for first internode. ** and *** denote significant at $P<0.05$ and $P<0.01$, respectively.

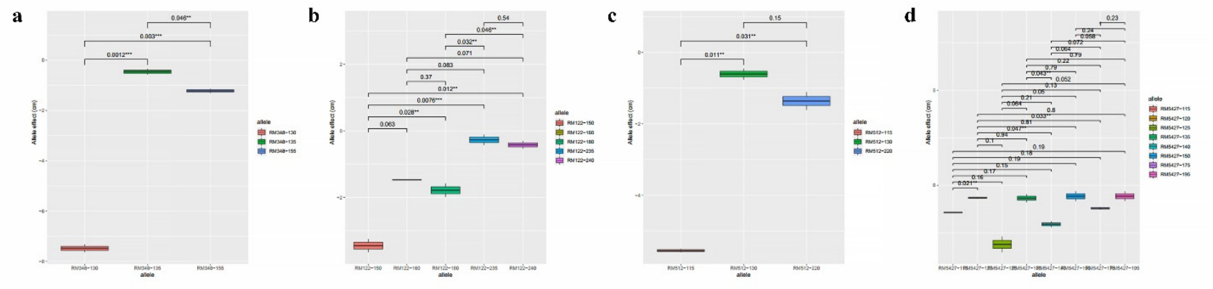

Figure S9. Pairwise *t* test for second internode. ** and *** denote significant at *P*<0.05 and *P*<0.01, respectively.

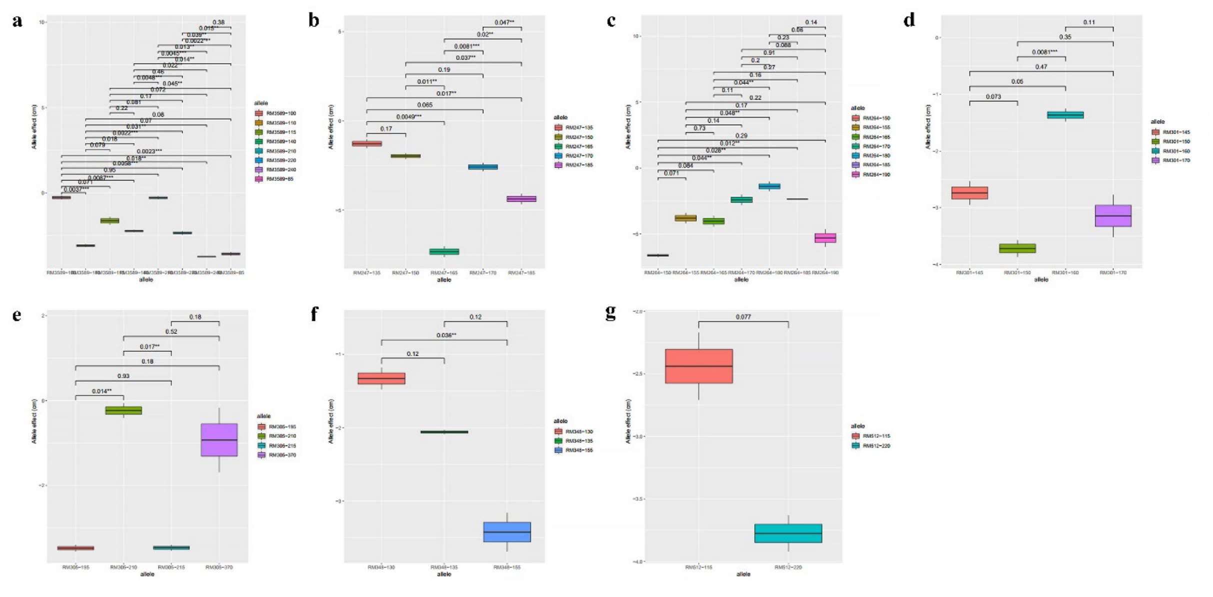

Figure S10. Pairwise *t* test for third internode. ** and *** denote significant at $P<0.05$ and $P<0.01$, respectively.

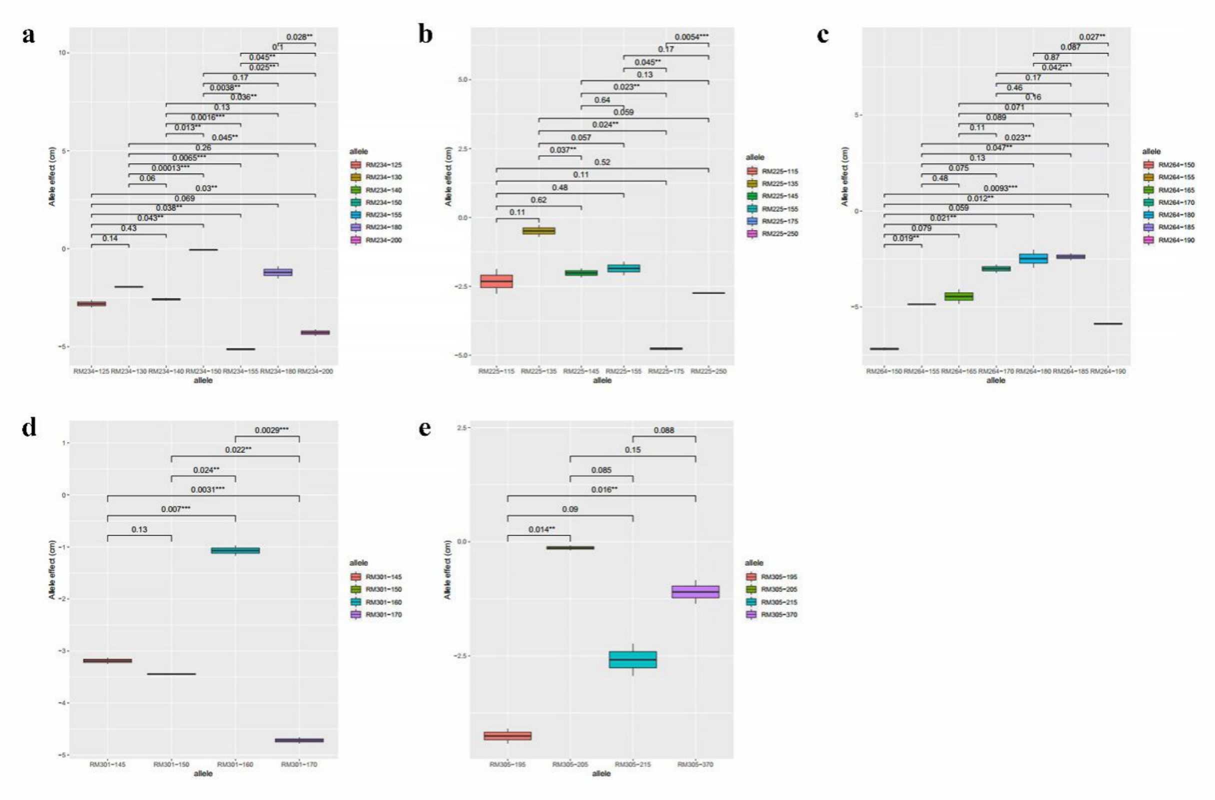

Figure S11. Pairwise *t* test for fourth internode. ** and *** denote significant at *P*<0.05 and *P*<0.01, respectively.

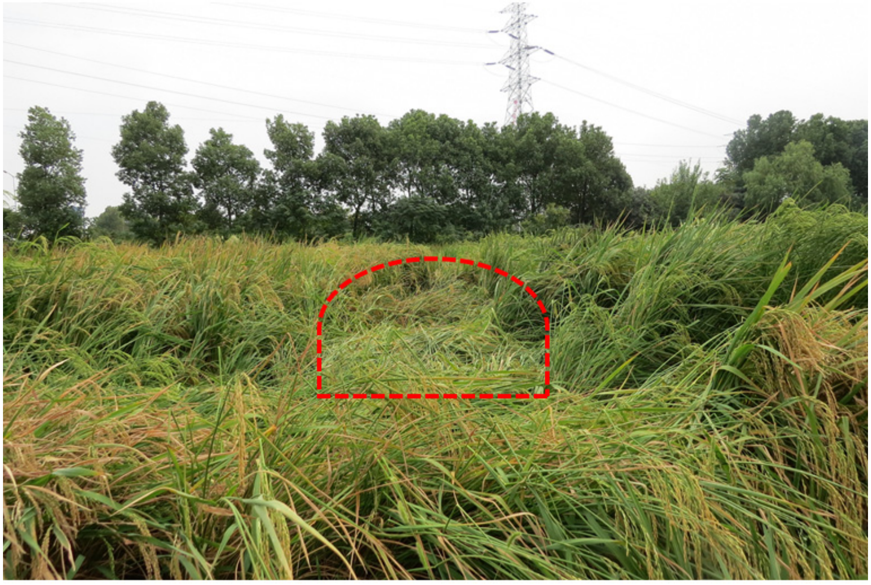

Figure S12. The scene of lodging in partial rice materials of natural population. The red dotted line represents the totally lodged materials among rice materials in natural population.

Table S1. Summary statistics for the 262 SSR markers used in the present study .

Marker	Chromosome	Position (cM)	Allele number	Gene diversity	Polymorphism information content
RM84	1	18.8	7	0.41	0.39
RM283	1	19.9	8	0.84	0.81
RM3453	1	25.4	13	0.85	0.84
RM1	1	25.4	11	0.8	0.78
RM259	1	29.7	21	0.89	0.88
RM583	1	38.8	6	0.71	0.66
RM490	1	43.2	5	0.28	0.27
RM8095	1	51	9	0.83	0.8
RM140	1	60.6	7	0.26	0.25
RM562	1	65.4	21	0.92	0.92
RM9	1	78.4	16	0.81	0.79
RM129	1	92.4	5	0.3	0.29
RM5	1	93	9	0.83	0.81
RM1231	1	98.5	16	0.9	0.9
RM128	1	123.2	15	0.85	0.84
RM297	1	126.5	9	0.77	0.73
RM246	1	132	14	0.86	0.85
RM212	1	134.6	5	0.39	0.37
RM5389	1	135.8	13	0.86	0.85
RM486	1	142.4	9	0.72	0.68
RM265	1	153.5	8	0.73	0.69
RM3482	1	155.9	14	0.85	0.83
RM6831	1	157.6	8	0.77	0.73
RM14	1	181.8	14	0.89	0.88
RM1-003	1	194	12	0.82	0.8
RM5340	2	36.3	16	0.88	0.87
RM7288	2	42.4	23	0.9	0.89
RM5356	2	43.3	10	0.85	0.83
RM1358	2	48.1	10	0.82	0.79
RM1313	2	51.1	10	0.81	0.78
RM324	2	51.1	5	0.41	0.37
RM327	2	51.9	9	0.83	0.81
RM301	2	53.5	8	0.77	0.74
RM300	2	54.6	15	0.88	0.86
RM262	2	70.2	9	0.78	0.75
RM5427	2	84.6	11	0.68	0.64
RM3688	2	88.2	8	0.86	0.84
RM183	2	93.5	14	0.86	0.84
RM5804	2	98.2	8	0.77	0.73

RM106	2	101.5	16	0.9	0.89
RM6361	2	102.9	8	0.74	0.7
RM573	2	118.1	15	0.84	0.83
RM450	2	122.8	7	0.81	0.79
RM7598	2	126.4	7	0.56	0.53
RM263	2	127.5	14	0.85	0.84
RM112	2	137.5	9	0.63	0.6
RM525	2	143.7	17	0.84	0.83
RM213	2	150.5	11	0.84	0.83
RM208	2	154.1	5	0.63	0.57
RM3850	2	156.3	14	0.85	0.83
RM498	2	156.3	13	0.81	0.79
RM48	2	191.2	8	0.42	0.4
RM266	2	192.2	10	0.75	0.72
RM535	2	195.7	16	0.87	0.86
RM132	3	3.9	5	0.4	0.38
RM1332	3	11.5	4	0.68	0.62
RM5849	3	18.4	15	0.82	0.79
RM489	3	20.3	14	0.89	0.88
RM545	3	24.7	5	0.59	0.52
RM5480	3	25.9	10	0.62	0.58
RM3467	3	28.2	14	0.89	0.88
RM3766	3	34.8	10	0.72	0.69
RM7	3	36.9	6	0.71	0.66
RM5639	3	39.8	7	0.72	0.68
RM7197	3	44.4	11	0.84	0.82
RM7345	3	48.8	7	0.65	0.61
RM282	3	55.8	11	0.84	0.82
RM338	3	61.9	8	0.75	0.71
RM218	3	67.8	9	0.71	0.67
RM232	3	76.7	11	0.83	0.81
RM7403	3	82.3	2	0.21	0.19
RM6266	3	94.9	6	0.62	0.58
RM7097	3	115.6	7	0.79	0.76
RM135	3	120.4	9	0.77	0.74
RM168	3	122.8	11	0.56	0.53
RM186	3	127.4	7	0.78	0.74
RM16	3	131.5	7	0.34	0.31
RM5475	3	137.9	17	0.9	0.89
RM416	3	140.1	9	0.72	0.68
RM6712	3	158.2	12	0.77	0.74
RM448	3	161.3	13	0.87	0.85
RM148	3	191.6	8	0.74	0.7

RM307	4	0	13	0.85	0.83
RM335	4	5.4	16	0.84	0.83
RM518	4	7.9	8	0.79	0.76
RM3471	4	16.7	19	0.89	0.88
RM4835	4	18.3	9	0.53	0.44
RM5687	4	25.4	9	0.76	0.73
RM6314	4	41.5	15	0.82	0.8
RM471	4	53.8	9	0.8	0.78
RM5951	4	56.1	5	0.69	0.64
RM142	4	60.2	15	0.87	0.86
RM6997	4	62.1	12	0.74	0.72
RM7563	4	68.3	8	0.84	0.83
RM6114	4	72	12	0.87	0.85
RM6589	4	85.2	8	0.83	0.81
RM317	4	96	6	0.71	0.66
RM6089	4	97.7	12	0.83	0.81
RM3513	4	99.6	7	0.53	0.49
RM3836	4	108.2	11	0.83	0.82
RM280	4	128.9	3	0.41	0.35
RM559	4	129.6	6	0.71	0.67
RM349	4	146.8	9	0.72	0.68
RM348	4	160.8	5	0.5	0.44
RM1182	5	3	11	0.85	0.83
RM153	5	3	13	0.83	0.81
RM122	5	3	6	0.25	0.23
RM159	5	5.4	14	0.89	0.88
RM267	5	25	12	0.85	0.84
RM437	5	31.5	2	0.26	0.22
RM3193	5	36.4	6	0.77	0.73
RM574	5	41	7	0.75	0.72
RM249	5	50.2	8	0.73	0.69
RM6082	5	53.5	15	0.84	0.83
RM598	5	62.7	6	0.52	0.48
RM473B	5	78.7	10	0.86	0.84
RM164	5	91.4	8	0.66	0.62
RM188	5	95.3	12	0.83	0.82
RM161	5	96.9	18	0.9	0.9
RM305	5	96.9	7	0.57	0.52
RM3170	5	115.4	13	0.87	0.86
RM480	5	130.6	17	0.88	0.87
RM5818	5	144.9	11	0.84	0.82
RM8109	6	1.7	10	0.77	0.74
RM508	6	2.3	13	0.83	0.81

RM510	6	11.5	11	0.83	0.81
RM225	6	26.2	12	0.75	0.72
RM405	6	28.6	6	0.73	0.69
RM2126	6	32.7	11	0.71	0.66
RM50	6	32.7	7	0.69	0.66
RM276	6	33.5	12	0.86	0.85
RM314	6	33.6	9	0.79	0.76
RM136	6	53	18	0.86	0.85
RM3330	6	61.6	14	0.89	0.88
RM3187	6	73.2	6	0.68	0.63
RM7579	6	84.5	6	0.74	0.69
RM8239	6	91.9	5	0.74	0.7
RM454	6	99.3	4	0.53	0.48
RM7309	6	100.3	15	0.86	0.84
RM528	6	100.8	11	0.74	0.7
RM3138	6	110.6	12	0.83	0.81
RM162	6	114.9	21	0.9	0.9
RM6811	6	115.6	15	0.9	0.89
RM345	6	123.9	7	0.6	0.55
RM5753	6	124.4	20	0.86	0.85
RM295	7	0	14	0.68	0.65
RM125	7	24.8	4	0.57	0.47
RM180	7	30.1	8	0.63	0.59
RM542	7	34.7	7	0.8	0.77
RM8263	7	35.7	8	0.67	0.62
RM418	7	42.1	9	0.72	0.68
RM346	7	47	9	0.77	0.74
RM2530	7	53.4	15	0.88	0.87
RM336	7	61	12	0.82	0.8
RM5380	7	67	10	0.78	0.74
RM6011	7	73.2	14	0.89	0.88
RM505	7	78.6	9	0.82	0.79
RM3589	7	89.8	13	0.82	0.8
RM11	7	93.8	8	0.77	0.74
RM234	7	93.9	14	0.8	0.78
RM134	7	99.6	10	0.83	0.81
RM1306	7	116.1	13	0.83	0.82
RM82	7	128.9	4	0.3	0.29
RM506	8	0	12	0.82	0.8
RM1019	8	0.5	17	0.87	0.86
RM152	8	9.4	13	0.8	0.78
RM1235	8	12.8	5	0.6	0.53
RM6863	8	16.4	11	0.79	0.76

RM4085	8	35.7	12	0.86	0.85
RM544	8	38.5	15	0.91	0.9
RM8243	8	50.8	11	0.88	0.87
RM25	8	52.2	12	0.78	0.75
RM331	8	59	10	0.8	0.77
RM72	8	60.9	14	0.81	0.79
RM6215	8	66.8	11	0.78	0.76
RM7556	8	86.7	12	0.84	0.82
RM6976	8	92.2	19	0.89	0.88
RM80	8	103.7	12	0.61	0.6
RM502	8	109.3	15	0.86	0.85
RM3754	8	112.6	7	0.81	0.79
RM6948	8	114.4	7	0.71	0.68
RM433	8	116	6	0.23	0.22
RM281	8	128.1	14	0.83	0.81
RM264	8	138.2	12	0.86	0.85
RM1328	9	0	15	0.88	0.87
RM8206	9	3.2	11	0.73	0.7
RM524	9	42.5	13	0.85	0.83
RM3912	9	46.3	9	0.85	0.83
RM566	9	50.7	9	0.76	0.73
RM434	9	57.7	9	0.86	0.85
RM3600	9	62.7	12	0.8	0.77
RM24481	9	63	19	0.9	0.9
RM3533	9	65.1	14	0.83	0.81
RM6570	9	68.2	9	0.53	0.5
RM410	9	79.3	9	0.69	0.64
RM257	9	79.7	11	0.77	0.74
RM201	9	81.2	9	0.77	0.74
OSR28	9	85.4	13	0.85	0.83
RM5384	9	90.7	10	0.86	0.84
RM1013	9	93.5	8	0.76	0.73
RM7492	10	0	12	0.87	0.86
RM7545	10	7.6	25	0.94	0.94
RM6646	10	13.3	9	0.82	0.8
RM244	10	15	6	0.48	0.45
RM216	10	24.8	3	0.61	0.54
RM311	10	25.2	8	0.8	0.78
RM184	10	41.6	5	0.73	0.69
RM1125	10	46.8	14	0.85	0.84
RM258	10	48.8	8	0.68	0.62
RM5629	10	53.6	11	0.77	0.75
RM6100	10	53.9	5	0.32	0.29

RM1108	10	55.3	6	0.29	0.27
RM3773	10	58.9	16	0.89	0.88
RM269	10	69.6	5	0.67	0.6
RM5352	10	71.4	5	0.52	0.43
RM304	10	73	6	0.13	0.13
RM171	10	73	4	0.6	0.52
RM6160	10	81	8	0.58	0.54
RM590	10	83.3	9	0.8	0.77
RM333	10	110.4	7	0.32	0.31
RM286	11	0.1	8	0.69	0.65
RM6327	11	1.7	15	0.88	0.87
RM1240	11	6.5	15	0.85	0.84
RM7557	11	9.2	5	0.58	0.53
RM1812	11	10.3	10	0.59	0.53
RM6544	11	19.8	5	0.46	0.42
RM3133	11	32.7	5	0.7	0.64
RM167	11	37.5	4	0.66	0.59
RM3701	11	45.3	10	0.73	0.69
RM7391	11	54.3	10	0.82	0.8
RM7303	11	64.2	4	0.09	0.09
RM7120	11	66.6	5	0.61	0.55
RM287	11	68.6	8	0.74	0.71
RM457	11	78.8	8	0.56	0.52
RM5349	11	79.1	6	0.66	0.6
RM209	11	84.7	6	0.4	0.37
RM21	11	85.7	11	0.77	0.73
RM7170	11	101.9	12	0.87	0.85
RM206	11	102.9	14	0.85	0.84
RM7163	11	112.4	2	0.08	0.08
RM6293	11	117.3	6	0.66	0.59
RM224	11	120.1	13	0.89	0.88
RM20	12	3.2	13	0.81	0.78
RM19	12	20.9	15	0.86	0.84
RM247	12	26.7	11	0.81	0.78
RM6296	12	26.7	5	0.56	0.52
RM7619	12	38.1	3	0.48	0.4
RM512	12	39.4	5	0.28	0.26
RM5746	12	39.4	16	0.86	0.85
RM277	12	48.2	3	0.4	0.33
RM1337	12	51.5	8	0.68	0.63
RM511	12	59.8	9	0.82	0.8
RM1246	12	65.3	10	0.68	0.65
RM7102	12	71.8	10	0.76	0.73

RM309	12	73	10	0.82	0.79
RM463	12	75.5	7	0.45	0.43
RM6869	12	75.8	15	0.87	0.86
RM3331	12	89.5	8	0.72	0.67
RM270	12	91.3	9	0.73	0.69
RM5479	12	95.4	16	0.72	0.7
RM17	12	107.4	11	0.85	0.83
RM12	12	107.4	4	0.54	0.45
**Total**			2664	190.62	183.23
**Mean**[a]			10.17	0.73	0.7

[a] computed by.

Table S2. The code, name and origin of 504 rice accessions divided into 8 sub populations corresponding to the probability value (Q value).

Code	Chinese name	Accession	Origin	Q values								POP
				Q1	Q2	Q3	Q4	Q5	Q6	Q7	Q8	
H18001	红芒沙粳	Hongmangzaodao	Kunshan, Jiangsu	0.001	0	0.001	0	0	0.088	0.909	0	Sub-pop 7
H18002	晚黄稻	Wanhuangdao	Wuxian, Jiangsu	0	0	0	0	0	0	0.999	0	Sub-pop 7
H18003	果子糯	Guozinuo	Kunshan, Jiangsu	0	0	0	0	0	0	0.999	0	Sub-pop 7
H18004	水晶白稻	Shuijingbaidao	Wuxian, Jiangsu	0	0	0	0	0	0	0.999	0	Sub-pop 7
H18005	无芒早稻	Wumangzaodao	Changshu, Jiangsu	0	0	0	0	0	0	0.999	0	Sub-pop 7
H18006	三百粒头	Sanbailitou	Kunshan, Jiangsu	0	0	0	0	0	0	0.999	0	Sub-pop 7
H18007	粗营晚洋稻	Cuyingwanyangdao	Wuxi, Jiangsu	0	0	0	0	0	0	0.999	0	Sub-pop 7
H18008	洋铃稻	Yanglingdao	Wuxi, Jiangsu	0	0	0	0	0	0	0.999	0	Sub-pop 7
H18009	敲冰黄	Qiaobinghuang	Taicang, Jiangsu	0	0	0	0	0	0	0.999	0	Sub-pop 7
H18010	铁粳青	Tiejingqing	Kunshan, Jiangsu	0	0	0	0	0	0	0.999	0	Sub-pop 7
H18011	小白野稻	Xiaobaiyedao	Wuxi, Jiangsu	0	0	0	0	0	0	0.999	0	Sub-pop 7
H18012	抱芯太湖青	Baoxintaihuqing	Wujiang, Jiangsu	0.005	0	0	0.001	0	0.005	0.988	0	Sub-pop 7
H18013	江丰 4 号	Jiangfeng4	Jiangyin, Jiangsu	0	0	0	0	0	0	0.999	0	Sub-pop 7
H18014	苏粳 4 号	Sujing4	Suzhou, Jiangsu	0	0	0	0	0	0	0.999	0	Sub-pop 7
H18015	矮种罗汉黄	Aizhongluohanhuang	Changshu, Jiangsu	0	0	0	0	0	0	1	0	Sub-pop 7
H18016	薄稻	Baodao	Wuxi, Jiangsu	0	0	0	0	0	0	1	0	Sub-pop 7
H18017	晚木樨球	Wanmuxiqiu	Taicang, Jiangsu	0	0	0	0	0	0	1	0	Sub-pop 7

H18018	荒三石	Huangsanshi	Wujiang, Jiangsu	0	0	0	0	0	0	1	0	Sub-pop 7
H18019	二黑稻	Erheidao	Wuxi, Jiangsu	0	0	0	0	0	0	1	0	Sub-pop 7
H18020	小青种	Xiaoqingzhong	Wuxian, Jiangsu	0	0	0	0	0	0	1	0	Sub-pop 7
H18021	早光头	Zaoguangtou	Wuxi, Jiangsu	0	0	0	0	0	0	1	0	Sub-pop 7
H18022	小罗汉黄	Xiaoluohanhuang	Changshu, Jiangsu	0	0	0	0	0	0	1	0	Sub-pop 7
H18023	苏州青	Souzhouqing	Jiangyin, Jiangsu	0	0	0	0	0	0	1	0	Sub-pop 7
H18024	晚芦栗	Wanluli	Jiangyin, Jiangsu	0	0	0	0	0	0	0.999	0	Sub-pop 7
H18025	晚八果	Wanbaguo	Jiangyin, Jiangsu	0	0	0	0	0	0	1	0	Sub-pop 7
H18026	老叠谷	Laodiegu	Wujiang, Jiangsu	0	0	0	0	0	0	0.999	0	Sub-pop 7
H18027	野凤凰	Yefenghuang	Wujiang, Jiangsu	0	0	0	0	0	0	1	0	Sub-pop 7
H18028	早黑头红	Zaoheitouhong	Wujiang, Jiangsu	0	0	0	0	0	0	1	0	Sub-pop 7
H18029	罗汉黄	Luohanhuang	Jiangyin, Jiangsu	0	0	0	0	0	0	1	0	Sub-pop 7
H18030	龙沟种	Longgouzhong	Qingpu, Shanghai	0	0	0	0	0	0	1	0	Sub-pop 7
H18031	石芦青	Shiluqing	Kunshan, Jiangsu	0	0	0	0	0	0	1	0	Sub-pop 7
H18032	立更青	Ligengqing	Yixing, Jiangsu	0	0	0	0	0	0	1	0	Sub-pop 7
H18033	早黑头红	Heitouhong	Wujiang, Jiangsu	0	0	0	0	0	0	1	0	Sub-pop 7
H18034	老来红	Laolaihong	Wuxian, Jiangsu	0	0	0	0	0	0	1	0	Sub-pop 7
H18035	二粒瘪	Erlibie	Wuxian, Jiangsu	0	0	0	0	0	0	0.999	0	Sub-pop 7
H18036	金谷黄	Jinguhuang	Wujiang, Jiangsu	0	0	0	0	0	0	0.999	0	Sub-pop 7
H18037	早十日黄稻	Zaoshirihuangdao	Wuxian, Jiangsu	0	0	0	0	0	0	0.999	0	Sub-pop 7

H18038	盛塘青	Shengtangqing	Changshu, Jiangsu	0	0	0	0	0	0	0.999	0	Sub-pop 7
H18039	小慢稻	Xiaomandao	Wujiang, Jiangsu	0	0	0	0	0	0	1	0	Sub-pop 7
H18040	盛塘青	Shengtangdao	Changshu, Jiangsu	0	0	0	0	0	0	1	0	Sub-pop 7
H18041	晚慢稻	Wanmandao	Wujiang, Jiangsu	0	0	0	0	0	0	1	0	Sub-pop 7
H18042	南头种	Nantouzhong	Kunshan, Jiangsu	0	0	0	0	0	0	1	0	Sub-pop 7
H18043	孔雀青	Kongqueqing	Kunshan, Jiangsu	0	0	0	0	0	0	0.999	0	Sub-pop 7
H18044	开青	Kaiqing	Kunshan, Jiangsu	0	0	0	0	0	0	0.999	0	Sub-pop 7
H18045	慢野稻	Manyedao	Kunshan, Jiangsu	0	0	0	0	0	0	0.999	0	Sub-pop 7
H18046	白壳糯	Baikenuo	Wujiang, Jiangsu	0	0	0	0	0	0	0.988	0.011	Sub-pop 7
H18047	白芒糯	Baimangnuo	Wujiang, Jiangsu	0	0.002	0	0	0	0.001	0.453	0.543	Admix
H18048	鸭血糯	Yaxienuo	Wuxian, Jiangsu	0	0	0	0	0	0	0.404	0.594	Admix
H18049	籼恢 429	Xianhui429	Nanjing, Jiangsu	0	0	0	0	0	0	0.479	0.52	Admix
H18050	紫尖籼 3	Zijianxian3	Nanjing, Jiangsu	0	0	0	0	0	0.027	0.353	0.619	Admix
H18051	荒三担糯	Huangsandannuo	Wuxi, Jiangsu	0	0	0	0	0	0	0.449	0.55	Admix
H18052	嘉 159	Jia159	Jiaxing, Zhejiang	0	0	0	0	0	0	0.456	0.543	Admix
H18053	泗稻 10 号	Sidao10	Siyang, Jiangsu	0	0	0	0	0	0	0.424	0.575	Admix
H18054	武羌	Wuqiang	Wujin, Jiangsu	0	0	0	0	0	0	0.361	0.638	Admix
H18055	武育粳 3 号	Wuyujing3	Wujin, Jiangsu	0	0	0	0	0	0.071	0.268	0.66	Admix
H18056	秀水 04	Xiushui04	Nanjing, Jiangsu	0	0.001	0	0	0	0.028	0.318	0.653	Admix
H18057	镇稻 88	Zhendao88	Zhenjiang, Jiangsu	0	0	0	0	0	0	0	0.999	Sub-pop 8

H18058	镇稻 6 号	Zhendao6	Zhenjiang, Jiangsu	0	0	0	0	0	0	0	0.999	Sub-pop 8
H18059	台粳 9 号选	Taijing9	Taibei,Tianwan	0	0	0	0	0	0	0	0.999	Sub-pop 8
H18060	台粳 16 号选低 AC	Taijing16xuanAC	Taibei,Tianwan	0	0	0	0	0	0	0	0.999	Sub-pop 8
H18061	台粳 16 号选紫	Taijing16xuanzi	Taibei,Tianwan	0	0	0	0	0	0	0	0.999	Sub-pop 8
H18062	H35(6435)	Hongdao35	Nanjing, Jiangsu	0	0	0	0	0	0	0	0.999	Sub-pop 8
H18063	H37(6427)	Hongdao37	Nanjing, Jiangsu	0	0	0	0	0	0	0	0.999	Sub-pop 8
H18064	南农粳 62401	Nannongjing62401	Nanjing, Jiangsu	0	0	0	0	0	0	0	0.999	Sub-pop 8
H18065	通粳 109	Tongjing109	Nantong, Jiangsu	0	0	0	0	0	0	0	0.999	Sub-pop 8
H18066	扬稻 6 号	Yangdao6	Yangzhou, Jiangsu	0	0	0	0	0	0	0	0.999	Sub-pop 8
H18067	宁粳 1 号	Nignjing1	Nanjing, Jiangsu	0	0	0	0	0	0	0	0.999	Sub-pop 8
H18068	武粳 15	Wujing15	Wujin, Jiangsu	0	0	0	0	0	0	0	1	Sub-pop 8
H18069	武香粳 14	Wuxiangjing14	Wujin, Jiangsu	0	0	0	0	0	0	0	0.999	Sub-pop 8
H18070	徐稻 3 号	Xudao3	Xuzhou, Jiangsu	0	0	0	0	0	0	0	0.999	Sub-pop 8
H18071	南农粳 003	Nannongjing003	Nanjing, Jiangsu	0	0	0	0	0	0	0	0.999	Sub-pop 8
H18072	南农粳 005	Nannongjing005	Nanjing, Jiangsu	0	0	0	0	0	0	0	0.999	Sub-pop 8
H18073	5 粳 20	5jing20	Nanjing, Jiangsu	0	0	0	0	0	0	0	0.999	Sub-pop 8
H18074	5 粳 15	5jing15	Nanjing, Jiangsu	0	0	0	0	0	0	0	1	Sub-pop 8
H18075	秣陵粳	molingjing	Nanjing, Jiangsu	0	0	0	0	0	0	0	1	Sub-pop 8
H18076	5 粳 03	5jing03	Nanjing, Jiangsu	0	0	0	0	0	0	0	1	Sub-pop 8
H18077	徐稻 4 号	Xudao4	Xuzhou, Jiangsu	0	0	0	0	0	0	0	1	Sub-pop 8

H18078	徐稻 5 号	Xudao5	Xuzhou, Jiangsu	0	0	0	0	0	0	0	1	Sub-pop 8
H18079	淮稻 9 号	Huaidao9	Huaian, Jiangsu	0	0	0	0	0	0	0	0.999	Sub-pop 8
H18080	盐稻 6 号	Yandao6	Yancheng, Jiangsu	0	0	0	0	0	0	0	1	Sub-pop 8
H18081	阳光 200	Yangguang200	Lianyungang, Jiangsu	0	0	0	0	0	0	0	0.999	Sub-pop 8
H18082	连粳 2 号	Lianjing2	Lianyungang, Jiangsu	0	0	0	0	0	0	0	0.999	Sub-pop 8
H18083	秀水 79（丙 8979）	Xiushui79	Jiaxing, Zhejiang	0	0	0	0	0	0	0	0.999	Sub-pop 8
H18084	C 堡	Cbao	Hefei, Anhui	0	0	0	0	0	0	0	1	Sub-pop 8
H18085	日本晴	Nipponbare	Japan	0	0	0	0	0	0	0	0.999	Sub-pop 8
H18086	镇 9424	Zhen9424	Zhenjiang, Jiangsu	0	0	0	0	0	0	0	1	Sub-pop 8
H18087	武育粳 7 号	Wuyujing7	Wujin, Jiangsu	0	0	0	0	0	0	0	0.999	Sub-pop 8
H18088	盐粳 8 号	Yanjing8	Yancheng, Jiangsu	0	0	0	0	0	0	0	0.999	Sub-pop 8
H18089	郑稻 18	Zhengdao18	Zhenzhou, Henan	0	0	0	0	0	0	0	0.999	Sub-pop 8
H18090	淮稻 11 号	Huaidao11	Huaian, Jiangsu	0	0.001	0	0	0	0	0.06	0.938	Sub-pop 8
H18091	圣稻 808	Shengdao808	Jiaxiang, Shandong	0	0.002	0	0	0	0	0.009	0.989	Sub-pop 8
H18092	圣稻 14	Shengdao14	Jiaxiang, Shandong	1	0	0	0	0	0	0	0	Admix
H18093	豫粳 6 号	Yujing6	Zhenzhou, Henan	0	0.001	0	0	0.261	0	0	0.738	Admix
H18094	淮稻 8 号	Huaidao8	Huaian, Jiangsu	0	0.721	0	0	0.278	0	0	0	Admix

H18095	津稻 12	Jindao12	Dongli, Tianjin	0	0.999	0	0	0	0	0	0	Sub-pop 2
H18096	皖稻 68	Wandao68	Hefei, Anhui	0	0.999	0	0	0	0	0	0	Sub-pop 2
H18097	宿粳 353	Sujing353	Suzhou, Jiangsu	0	0.999	0	0	0	0	0	0	Sub-pop 2
H18098	香粳 9407	Xiangjing9407	Nanjing, Jiangsu	0	0.999	0	0	0	0	0	0	Sub-pop 2
H18099	中粳 212	Zhongjing212	Nanjing, Jiangsu	0	0.999	0	0	0	0	0	0	Sub-pop 2
H18100	中粳 9677	Zhongjing9677	Nanjing, Jiangsu	0	0.999	0	0	0	0	0	0	Sub-pop 2
H18101	中粳 131	Zhongjing131	Nanjing, Jiangsu	0	0.999	0	0	0	0	0	0	Sub-pop 2
H18102	中粳 438	Zhognjing438	Nanjing, Jiangsu	0	0.999	0	0	0	0	0	0	Sub-pop 2
H18103	盐粳 9 号	Yanjing9	Yancheng, Jiangsu	0	0.999	0	0	0	0	0	0	Sub-pop 2
H18104	扬辐粳 4901	Yangfujing4901	Yangzhou, Jiangsu	0	0.999	0	0	0	0	0	0	Sub-pop 2
H18105	紫粳	Zijing	Nanjing, Jiangsu	0	0.999	0	0	0	0	0	0	Sub-pop 2
H18106	镇稻 10 号	Zhendao10	Zhenjiang, Jiangsu	0	0.999	0	0	0	0	0	0	Sub-pop 2
H18107	郑旱 2 号	Zhenghan2	Zhengzhou, Henan	0	0.999	0	0	0	0	0	0	Sub-pop 2
H18108	郑旱 6 号	Zhen6	Zhengzhou, Henan	0	0.999	0	0	0	0	0	0	Sub-pop 2
H18109	西石黄	Xishihuang	Wuxian, Jiangsu	0	0.999	0	0	0	0	0	0	Sub-pop 2
H18110	大量稻	Daliangdao	Wuxi, Jiangsu	0	0.999	0	0	0	0	0	0	Sub-pop 2
H18111	黑嘴稻	Heizuidao	Changshu, Jiangsu	0	1	0	0	0	0	0	0	Sub-pop 2
H18112	小黄稻	Xiaohuangdao	Wuxian, Jiangsu	0	0.999	0	0	0	0	0	0	Sub-pop 2
H18113	凤凰稻	Fenghaungdao	Changshu, Jiangsu	0	0.999	0	0	0	0	0	0	Sub-pop 2

H18114	麦节青	Maijieqing	Songjiang, Shanghai	0	0.999	0	0	0	0	0	0	Sub-pop 2
H18115	鸡粳稻	Jijingdao	Wujiang, Jiangsu	0	0.999	0	0	0	0	0	0	Sub-pop 2
H18116	中熟洋种稻	Zhognsuyangzhogndao	Wuxi, Jiangsu	0	0.999	0	0	0	0	0	0	Sub-pop 2
H18117	堆谷种	Duiguzhong	Wujiang, Jiangsu	0	0.999	0	0	0	0	0	0	Sub-pop 2
H18118	甩杀板	Shuaishaban	Songjiang, Shanghai	0	0.999	0	0	0	0	0	0	Sub-pop 2
H18119	牛毛黄	Niumaohuang	Taicang, Jiangsu	0	1	0	0	0	0	0	0	Sub-pop 2
H18120	晚黑头红	Wanheitouhong	Wujiang, Jiangsu	0	0.999	0	0	0	0	0	0	Sub-pop 2
H18121	太湖青	Taihuqing	Kunshan, Jiangsu	0	0.999	0	0	0	0	0	0	Sub-pop 2
H18122	一粒芒	Yilimang	Changshu, Jiangsu	0	0.999	0	0	0	0	0	0	Sub-pop 2
H18123	五齐头	Wuqitou	Wujin, Jiangsu	0	0.999	0	0	0	0	0	0	Sub-pop 2
H18124	脚矮黑头红	Jiaoaiheitouhong	Wujiang, Jiangsu	0	0.999	0	0	0	0	0	0	Sub-pop 2
H18125	老勿死	Laowusi	Wujiang, Jiangsu	0	0.999	0	0	0	0	0	0	Sub-pop 2
H18126	慢绿种	Manliuzhong	Jinshan, Shanghai	0	0.999	0	0	0	0	0	0	Sub-pop 2
H18127	天落黄	Tainluohuang	Changshu, Jiangsu	0	1	0	0	0	0	0	0	Sub-pop 2
H18128	韭菜青	Jiucaiqing	Changshu, Jiangsu	0	0.999	0	0	0	0	0	0	Sub-pop 2
H18129	矮萁大绿种	Aiqidaliuzhong	Jiading, Shanghai	0	0.999	0	0	0	0	0	0	Sub-pop 2
H18130	芦粳青	Lujingqing	Wujiang, Jiangsu	0	0.999	0	0	0	0	0	0	Sub-pop 2
H18131	高粱青	Gaoliangqing	Wujiang, Jiangsu	0	0.999	0	0	0	0	0	0	Sub-pop 2
H18132	一时兴	Yishixing	Changshu, Jiangsu	0	0.999	0	0	0	0	0	0	Sub-pop 2

H18133	黑种	Heizhong	Wuxian, Jiangsu	0	0.999	0	0	0	0	0	0	Sub-pop 2
H18134	雪里青	Xueliqing	Wuxi, Jiangsu	0	0.999	0	0	0	0	0	0	Sub-pop 2
H18135	溧阳小红稻	Liyangxiaohongdao	Liyang, Jiangsu	0	0.999	0	0	0	0	0	0	Sub-pop 2
H18136	菊花黄	Juhuahuang	Wuxi, Jiangsu	0	0.999	0	0	0	0	0	0	Sub-pop 2
H18137	长子粳野稻	Changzijingyedao	Wuxian, Jiangsu	0	0.999	0	0	0	0	0	0	Sub-pop 2
H18138	盖稻青	Gaidaoqing	Wujiang, Jiangsu	0	0.999	0	0	0	0	0	0	Sub-pop 2
H18139	丁庄稻	Dingzhuangdao	Wuxi, Jiangsu	0	0.999	0	0	0	0	0	0	Sub-pop 2
H18140	学堂种	Xuetangzhong	Jiangyin, Jiangsu	0	0.999	0	0	0	0	0	0	Sub-pop 2
H18141	掼百担	Guanbaidan	Wujiang, Jiangsu	0	0	0.999	0	0	0	0	0	Sub-pop 3
H18142	红薄稻	Hongbaodao	Jiaxing, Zhejiang	0	0	0.999	0	0	0	0	0	Sub-pop 3
H18143	铁秆稻	Tiegandao	Wujiang, Jiangsu	0	0	0.999	0	0	0	0	0	Sub-pop 3
H18144	聚子光	Juzigaung	Wuxi, Jiangsu	0	0	0.999	0	0	0	0	0	Sub-pop 3
H18145	野白稻	Yebaidao	Taicang, Jiangsu	0	0	0.999	0	0	0	0	0	Sub-pop 3
H18146	大黑头红	Daheitouhong	Wujiang, Jiangsu	0	0	0.999	0	0	0	0	0	Sub-pop 3
H18147	百歌稻	Baigedao	Wuxian, Jiangsu	0	0	0.857	0	0	0.142	0	0	Admix
H18148	叠叠种	Diediezhong	Qingpu, Shanghai	0	0	0.999	0	0	0	0	0	Sub-pop 3
H18149	浦西大稻头	Puxidadaotou	Wujiang, Jiangsu	0	0	0.999	0	0	0	0	0	Sub-pop 3
H18150	洋稻	Yangdao	Wujiang, Jiangsu	0	0	0.999	0	0	0	0	0	Sub-pop 3
H18151	晏红稻	Yanhongdao	Wujiang, Jiangsu	0	0	0.999	0	0	0	0	0	Sub-pop 3
H18152	白壳晚稻	Baikewandao	Wuxi, Jiangsu	0	0	0.999	0	0	0	0	0	Sub-pop 3

H18153	爱国大稻头	Aiguodadaotou	Wujiang, Jiangsu	0	0	0.96	0	0	0.039	0	0	Sub-pop 3
H18154	四石头	Sishitou	Wuxian, Jiangsu	0	0.001	0.999	0	0	0	0	0	Sub-pop 3
H18155	千斤稻	Qianjindao	Wujiang, Jiangsu	0	0	0.999	0	0	0	0	0	Sub-pop 3
H18156	柏树青	Baishuqing	Qingpu, Shanghai	0	0	0.999	0	0	0	0	0	Sub-pop 3
H18157	嘉 45	Jia45	Jiaxing, Zhejiang	0	0	0.57	0.43	0	0	0	0	Admix
H18158	秋田大泻稻	Qiutiandaxiedao	Wujiang, Jiangsu	0	0	0.999	0	0	0	0	0	Sub-pop 3
H18159	琦玉糯 10	Qiyunuo10	Wujiang, Jiangsu	0	0	0.999	0	0	0	0	0	Sub-pop 3
H18160	吴糯一号	Wunuo1	Wujiang, Jiangsu	0	0	0.999	0	0	0	0	0	Sub-pop 3
H18161	加农糯 2 号	Jianongnuo2	Wujiang, Jiangsu	0	0	0.999	0	0	0	0	0	Sub-pop 3
H18162	红农 5 号	Hongnong5	Wujiang, Jiangsu	0	0	0.999	0	0	0	0	0	Sub-pop 3
H18163	农林糯 4 号	Nonglinnuo4	Wujiang, Jiangsu	0	0	0.999	0	0	0	0	0	Sub-pop 3
H18164	香糯稻	Xiangnuodao	Wuxian, Jiangsu	0	0	0.999	0	0	0	0	0	Sub-pop 3
H18165	芦柴红	Luchaihong	Wujiang, Jiangsu	0	0	0.999	0	0	0	0	0	Sub-pop 3
H18166	寸谷	Cungu	Wujiang, Jiangsu	0	0	0.999	0	0	0	0	0	Sub-pop 3
H18167	卡特纳	Katena	Wujiang, Jiangsu	0	0	0.999	0	0	0	0	0	Sub-pop 3
H18168	掼刹糯	Guanchanuo	Wujiang, Jiangsu	0	0	0.999	0	0	0	0	0	Sub-pop 3
H18169	葵花糯	Kuihuanuo	Wuxian, Jiangsu	0	0	0.999	0	0	0	0	0	Sub-pop 3
H18170	苏御糯	Suyunuo	Wuxian, Jiangsu	0	0	0.999	0	0	0	0	0	Sub-pop 3
H18171	红脚占	Hongjiaozhan	Wuxian, Jiangsu	0	0	0.999	0	0	0	0	0	Sub-pop 3
H18172	毫补卡	Haobuka	Wuxian, Jiangsu	0	0	0.999	0	0	0	0	0	Sub-pop 3

H18173	出阳旱 32	Chuyanghan32	Wuxian, Jiangsu	0	0	0.999	0	0	0	0	0	Sub-pop 3
H18174	利班一号	Libanyi	Wuxian, Jiangsu	0	0	0.999	0	0	0	0	0	Sub-pop 3
H18175	昆农 8 号	Kunnong8	Kunshan, Jiangsu	0	0	0.999	0	0	0	0	0	Sub-pop 3
H18176	桂花黄	Guihuahuang	Nanjing, Jiangsu	0	0	0.988	0	0	0.011	0	0	Sub-pop 3
H18177	周家种	Zhoujiazhong	Wujiang, Jiangsu	0	0	0.999	0	0	0	0	0	Sub-pop 3
H18178	小凤凰	Xiaofenghuang	Wuxian, Jiangsu	0	0	0.999	0	0	0	0	0	Sub-pop 3
H18179	香粳稻	Xiangjingdao	Wuxian, Jiangsu	0	0	0.971	0	0	0.028	0	0	Sub-pop 3
H18180	灰藻	Huizao	Wujiang, Jiangsu	0	0	0.999	0	0	0	0	0	Sub-pop 3
H18181	硬头稻	Yingtoudao	Kunshan, Jiangsu	0.001	0	0.927	0	0	0.071	0	0	Sub-pop 3
H18182	长稻头	Changdaotou	Wujiang, Jiangsu	0	0	0.892	0	0	0.106	0.001	0	Admix
H18183	杨庙种	Yangmiaozhong	Wujiang, Jiangsu	0	0	0.915	0	0	0.084	0	0	Sub-pop 3
H18184	毛光稻	Maoguangdao	Wuxian, Jiangsu	0	0	0.943	0	0	0.056	0	0	Sub-pop 3
H18185	大种稻	Dazhongdao	Wujiang, Jiangsu	0	0	0.999	0	0	0	0	0	Sub-pop 3
H18186	三吓稻	Sanxiadao	Wuxi, Jiangsu	0	0	0.999	0	0	0	0	0	Sub-pop 3
H18187	小青芒	Xiaoqingmang	Changshu, Jiangsu	0	0	0.999	0	0	0	0	0	Sub-pop 3
H18188	红秆荔枝红	Hongganlizhihong	Wujiang, Jiangsu	0	0	0.999	0	0	0	0	0	Sub-pop 3
H18189	无锡稻	Wuxidao	Changshu, Jiangsu	0	0	0.999	0	0	0	0	0	Sub-pop 3
H18190	晚中秋	Wanzhognqiu	Wuxian, Jiangsu	0	0	0.999	0	0	0	0	0	Sub-pop 3
H18191	风景稻	Fengjingdao	Wuxian, Jiangsu	0	0	0.999	0	0	0	0	0	Sub-pop 3
H18192	绿种	Liuzhong	Changshu, Jiangsu	0	0	0.999	0	0	0	0	0	Sub-pop 3

H18193	粗秆荔枝红	Cuganlizhihong	Wuxian, Jiangsu	0	0	0.999	0	0	0	0	0	Sub-pop 3
H18194	赤谷晚稻	Chiguwandao	Wujiang, Jiangsu	0	0	0.999	0	0	0	0	0	Sub-pop 3
H18195	茭白叶青	Jiaobaiyeqing	Songjiang, Shanghai	0	0	0.999	0	0	0	0	0	Sub-pop 3
H18196	迟谷红	Chiguhong	Wujiang, Jiangsu	0	0	0.999	0	0	0	0	0	Sub-pop 3
H18197	饭箩青	Fanluoqing	Kunshan, Jiangsu	0	0	0.999	0	0	0	0	0	Sub-pop 3
H18198	早野稻	Zaoyedao	Kunshan, Jiangsu	0	0	0.999	0	0	0	0	0	Sub-pop 3
H18199	白叠谷	Baidiegu	Wujiang, Jiangsu	0	0	0.999	0	0	0	0	0	Sub-pop 3
H18200	王家稻	Wangjiadao	Wujiang, Jiangsu	0	0	0.999	0	0	0	0	0	Sub-pop 3
H18201	江阴种	Jiangyinzhong	Jiangyin, Jiangsu	0	0	0.999	0	0	0	0	0	Sub-pop 3
H18202	鹅营白粳稻	Eyingbaijingdao	Jiading, Shanghai	0	0	0.999	0	0	0	0	0	Sub-pop 3
H18203	铁壳晚光头	Tiekewanguangtou	Wujin, Jiangsu	0	0	0.999	0	0	0	0	0	Sub-pop 3
H18204	铁壳稻	Tiekedao	Wujin, Jiangsu	0	0	0.999	0	0	0	0	0	Sub-pop 3
H18205	大稻穗头	Dadaosuitou	Changshu, Jiangsu	0	0	0.915	0	0	0.084	0	0	Sub-pop 3
H18206	矮白稻	Aibaidao	Wujiang, Jiangsu	0	0	0.999	0	0	0	0	0	Sub-pop 3
H18207	小白稻	Xiaobaidao	Wuxian, Jiangsu	0	0	0.999	0	0	0	0	0	Sub-pop 3
H18208	白石稻	Baishidao	Taicang, Jiangsu	0	0	0.999	0	0	0	0	0	Sub-pop 3
H18209	慢白稻	Manbaidao	Wujiang, Jiangsu	0	0	0.999	0	0	0	0	0	Sub-pop 3
H18210	光头芦花白	Guangtouluhuabai	Wuxi, Jiangsu	0	0	0.999	0	0	0	0	0	Sub-pop 3
H18211	红芒粳	Hongmangjing	Kunshan, Jiangsu	0	0	0.999	0	0	0	0	0	Sub-pop 3
H18212	无芒野稻	Wumangyedao	Changshu, Jiangsu	0	0	0.999	0	0	0	0	0	Sub-pop 3

H18213	芦花白	Luhuabai	Wuxian, Jiangsu	0	0	0.999	0	0	0	0	0	Sub-pop 3
H18214	海冬青	Haidongqing	Kunshan, Jiangsu	0	0	0.999	0	0	0	0	0	Sub-pop 3
H18215	神乐糯	Shenlenuo	Kunshan, Jiangsu	0	0	0.999	0	0	0	0	0	Sub-pop 3
H18216	湘晴	Xiangqing	Chongming, Shanghai	0	0	0.999	0	0	0	0	0	Sub-pop 3
H18217	粳恢 418	Jinghui418	Shenyang, Liaoning	0	0	0.999	0	0	0	0	0	Sub-pop 3
H18218	马来红	Malaihong	Nanjing, Jiangsu	0	0	0.999	0	0	0	0	0	Sub-pop 3
H18219	粳糯 330	Jingnuo330	Hefei, Anhui	0	0	0.999	0	0	0	0	0	Sub-pop 3
H18220	再进粳	Zaijinjing	Huaian, Jiangsu	0	0	0.999	0	0	0	0	0	Sub-pop 3
H18221	富玉 3 号	Fuyu3	Yuexi, Anhui	0	0	0.999	0	0	0	0	0	Sub-pop 3
H18222	R254	R254	Chongming, Shanghai	0	0	0.999	0	0	0	0	0	Sub-pop 3
H18223	江阴糯	Jiangyinnuo	Jiangyin, Jiangsu	0	0	0.999	0	0	0	0	0	Sub-pop 3
H18224	粳谷糯	Jinggunuo	Wuxi, Jiangsu	0	0	0.999	0	0	0	0	0	Sub-pop 3
H18225	单红谷	Shanhonggu	Wujiang, Jiangsu	0	0	0.999	0	0	0	0	0	Sub-pop 3
H18226	晚生毛黄	Wanshengmaohuang	Wuxi, Jiangsu	0	0	0.996	0	0	0.003	0	0	Sub-pop 3
H18227	晚洋稻	Wanyangdao	Wuxian, Jiangsu	0	0	0.957	0	0	0.042	0	0	Sub-pop 3
H18228	矮大种	Aidazhong	Wujiang, Jiangsu	0	0	0.862	0	0	0.137	0	0	Admix
H18229	鸡脚红	Jijiaohong	Wuxian, Jiangsu	0	0	0.101	0.898	0	0	0	0	Admix
H18230	偷来种	Toulaizhong	Wujiang, Jiangsu	0	0	0.146	0.853	0	0	0	0	Admix

H18231	花壳诺	Huakenuo	Wujiang, Jiangsu	0	0	0.001	0.999	0	0	0	0	Admix
H18232	头等一时兴	Toudengyishixing	Kunshan, Jiangsu	0	0	0	1	0	0	0	0	Sub-pop 4
H18233	帽子头	Maozitou	Wujiang, Jiangsu	0	0.001	0.013	0.985	0	0	0	0	Sub-pop 4
H18234	早糯稻	Zaonuodao	Wujiang, Jiangsu	0	0	0.01	0.989	0	0	0	0	Sub-pop 4
H18235	大头鬼	Datougui	Changshu, Jiangsu	0	0.001	0.002	0.996	0	0	0	0	Sub-pop 4
H18236	早小白稻	Zaoxiaobaidao	Wuxi, Jiangsu	0	0	0.007	0.992	0	0	0	0	Sub-pop 4
H18237	杭州糯	Kangzhounuo	Wujiang, Jiangsu	0	0	0.002	0.997	0	0	0	0	Sub-pop 4
H18238	阔板种	Kuobanzhong	Qingpu, Shanghai	0	0	0.065	0.934	0	0	0	0	Sub-pop 4
H18239	黄壳晚光头	Huangkewanguangtou	Wujin, Jiangsu	0	0	0.078	0.921	0	0	0	0	Sub-pop 4
H18240	铁捍一时兴	Tiehanyishixing	Wuxi, Jiangsu	0	0.001	0.008	0.99	0	0	0	0	Sub-pop 4
H18241	矮脚芦秆黄	Aijiaoluganhuang	Changshu, Jiangsu	0	0	0	1	0	0	0	0	Sub-pop 4
H18242	中花 3 号	Zhonghua3	Haidian, Beijing	0	0	0	1	0	0	0	0	Sub-pop 4
H18243	补血糯	Buxienuo	kunshan, Jiangsu	0	0	0	1	0	0	0	0	Sub-pop 4
H18244	皖粳糯	Wanjingnuo	Hefei, Anhui	0	0	0	1	0	0	0	0	Sub-pop 4
H18245	C418	C418	Shenyang, Liaoning	0	0	0	1	0	0	0	0	Sub-pop 4
H18246	富香 1 号	Fuxiang1	Yuexi, Anhui	0	0	0	1	0	0	0	0	Sub-pop 4
H18247	越 1（BT1）	Yue1（BT1）	Vietnam	0	0	0	1	0	0	0	0	Sub-pop 4
H18248	越 2（ML203）	Yue2（ML203）	Vietnam	0	0	0	1	0	0	0	0	Sub-pop 4
H18249	越 3(2490)	Yue3(2490)	Vietnam	0	0	0	1	0	0	0	0	Sub-pop 4
H18250	越 4[88-6(HH)3]	Yue4[88-6(HH)3]	Vietnam	0	0	0	1	0	0	0	0	Sub-pop 4

H18251	越 5(KD)	Yue5(KD)	Vietnam	0	0	0	1	0	0	0	0	Sub-pop 4
H18252	越 6(N202)	Yue6(N202)	Vietnam	0	0	0	1	0	0	0	0	Sub-pop 4
H18253	越 8(VD108-1)	Yue8(VD108-1)	Vietnam	0	0	0	1	0	0	0	0	Sub-pop 4
H18254	越 9(KD18)	Yue9(KD18)	Vietnam	0	0	0	1	0	0	0	0	Sub-pop 4
H18255	越 10(DB5)	Yue10(DB5)	Vietnam	0	0	0	1	0	0	0	0	Sub-pop 4
H18256	越 11(VD7)	Yue11(VD7)	Vietnam	0	0	0	1	0	0	0	0	Sub-pop 4
H18257	越 12(OM2718)	Yue12(OM2718)	Vietnam	0	0	0	1	0	0	0	0	Sub-pop 4
H18258	越 13(AYTO1)	Yue13(AYTO1)	Vietnam	0	0	0	1	0	0	0	0	Sub-pop 4
H18259	越 14(KDDB)	Yue14(KDDB)	Vietnam	0	0	0	0.999	0	0	0	0	Sub-pop 4
H18260	越 15(OM3003)	Yue15(OM3003)	Vietnam	0	0	0	0.999	0	0	0	0	Sub-pop 4
H18261	越 16(DB6)	Yue16(DB6)	Vietnam	0	0	0	0.999	0	0	0	0	Sub-pop 4
H18262	越 17(ML202)	Yue17(ML202)	Vietnam	0	0	0	0.999	0	0	0	0	Sub-pop 4
H18263	越 18(AYTO1-D12)	Yue18(AYTO1-D12)	Vietnam	0	0	0	0.999	0	0	0	0	Sub-pop 4
H18264	越 19(AYTO1-D12)	Yue19(AYTO1-D12)	Vietnam	0	0	0	0.999	0	0	0	0	Sub-pop 4
H18265	越 20(OM1490)	Yue20(OM1490)	Vietnam	0	0	0	0.999	0	0	0	0	Sub-pop 4
H18266	越 21(94-3-1)	Yue21(94-3-1)	Vietnam	0	0	0	0.999	0	0.001	0	0	Sub-pop 4
H18267	越 22(LCV22)	Yue22(LCV22)	Vietnam	0	0	0	0.999	0	0	0	0	Sub-pop 4
H18268	越 23(LCV10)	Yue23(LCV10)	Vietnam	0	0	0	0.999	0	0	0	0	Sub-pop 4
H18269	越 24（LCV18）	Yue24（LCV18）	Vietnam	0	0	0	0.999	0	0	0	0	Sub-pop 4

H18270	越 25（121）	Yue25（121）	Vietnam	0	0	0	0.999	0	0	0	0	Sub-pop 4
H18271	越 26（75）	Yue26（75）	Vietnam	0	0	0	0.999	0	0	0	0	Sub-pop 4
H18272	越 27（52）	Yue27（52）	Vietnam	0	0	0	0.999	0	0	0	0	Sub-pop 4
H18273	越 28（49）	Yue28（49）	Vietnam	0	0	0	0.999	0	0	0	0	Sub-pop 4
H18274	越 29（3）	Yue29（3）	Vietnam	0	0	0	0.999	0	0	0	0	Sub-pop 4
H18275	越 30（3）	Yue30（3）	Vietnam	0	0	0	0.999	0	0	0	0	Sub-pop 4
H18276	越 31（45）	Yue31（45）	Vietnam	0	0	0	0	0	0.999	0	0	Sub-pop 6
H18277	越 32[16(10)]	Yue32[16(10)]	Vietnam	0	0	0	0	0	0.999	0	0	Sub-pop 6
H18278	越 33(8603)	Yue33(8603)	Vietnam	0	0	0	0	0	0.999	0	0	Sub-pop 6
H18279	越 34(15.9-4)	Yue34(15.9-4)	Vietnam	0	0	0	0	0	0.999	0	0	Sub-pop 6
H18280	越 35(53)	Yue35(53)	Vietnam	0	0	0	0	0	0.999	0	0	Sub-pop 6
H18281	越 36(17.10-1)	Yue36(17.10-1)	Vietnam	0	0	0	0	0	0.999	0	0	Sub-pop 6
H18282	越 37(2)	Yue37(2)	Vietnam	0	0	0	0	0	0.999	0	0	Sub-pop 6
H18283	越 38(70)	Yue38(70)	Vietnam	0	0	0	0	0	0.999	0	0	Sub-pop 6
H18284	越 39(34)	Yue39(34)	Vietnam	0	0	0	0	0	0.999	0	0	Sub-pop 6
H18285	越 41(47)	Yue41(47)	Vietnam	0	0	0	0	0	1	0	0	Sub-pop 6
H18286	越 42(85)	Yue42(85)	Vietnam	0	0	0	0	0	0.999	0	0	Sub-pop 6
H18287	越 43(59)	Yue43(59)	Vietnam	0	0	0	0	0	0.999	0	0	Sub-pop 6
H18288	越 44(59)	Yue44(59)	Vietnam	0	0	0	0	0	0.999	0	0	Sub-pop 6
H18289	越 45(PC5)	Yue45(PC5)	Vietnam	0	0	0	0	0	0.999	0	0	Sub-pop 6

H18290	越 46(PC6)	Yue46(PC6)	Vietnam	0	0	0	0	0	0.999	0	0	Sub-pop 6
H18291	越 47(HT1)	Yue47(HT1)	Vietnam	0	0	0	0	0	0.999	0	0	Sub-pop 6
H18292	越 48(D6)	Yue48(D6)	Vietnam	0	0	0	0	0	0.999	0	0	Sub-pop 6
H18293	越 49(AC5)	Yue49(AC5)	Vietnam	0	0	0	0	0	0.999	0	0	Sub-pop 6
H18294	越 50(KHAO 85)	Yue50(KHAO 85)	Vietnam	0	0	0	0	0	0.999	0	0	Sub-pop 6
H18295	越 52（5028）	Yue52（5028）	Vietnam	0	0	0	0	0	0.999	0	0	Sub-pop 6
H18296	越 54（B0T1）	Yue54（B0T1）	Vietnam	0	0	0	0	0	0.999	0	0	Sub-pop 6
H18297	越 56（IR64）	Yue56（IR64）	Vietnam	0	0	0	0	0	1	0	0	Sub-pop 6
H18298	越 57（254）	Yue57（254）	Vietnam	0	0	0	0	0	0.999	0	0	Sub-pop 6
H18299	越 58（T10）	Yue58（T10）	Vietnam	0	0	0	0	0	0.999	0	0	Sub-pop 6
H18300	越 59（BT7 中国）	Yue59（BT7China）	Vietnam	0	0	0	0	0	0.999	0	0	Sub-pop 6
H18301	越 60（PC8)	Yue60（PC8)	Vietnam	0	0	0	0	0	0.999	0	0	Sub-pop 6
H18302	越 61(D19)	Yue61(D19)	Vietnam	0	0	0	0	0	0.999	0	0	Sub-pop 6
H18303	越 62(D28)	Yue62(D28)	Vietnam	0	0	0	0	0	0.999	0	0	Sub-pop 6
H18304	越 63(VN2002-1)	Yue63(VN2002-1)	Vietnam	0	0	0	0	0	0.999	0	0	Sub-pop 6
H18305	越 64(DSDL 台湾特色)	Yue64(DSDL Taiwan Features)	Vietnam	0	0	0	0	0	0.999	0	0	Sub-pop 6
H18306	越 66(CL9)	Yue66(CL9)	Vietnam	0	0	0	0	0	0.999	0	0	Sub-pop 6
H18307	越 68(PC10)	Yue68(PC10)	Vietnam	0	0	0	0	0	0.999	0	0	Sub-pop 6
H18308	越 69(3486)	Yue69(3486)	Vietnam	0	0	0	0	0	0.999	0	0	Sub-pop 6

H18309	越 70(PC7)	Yue70(PC7)	Vietnam	0	0	0	0	0	0.999	0	0	Sub-pop 6
H18310	越 71(N2-08)	Yue71(N2-08)	Vietnam	0	0	0	0	0	0.999	0	0	Sub-pop 6
H18311	越 72(N1-08)	Yue72(N1-08)	Vietnam	0	0	0	0.413	0	0.586	0	0	Admix
H18312	越 73(GIA LOC 6)	Yue73(GIA LOC 6)	Vietnam	0	0	0.001	0	0.001	0.997	0	0	Sub-pop 6
H18313	越 74(N4-05)	Yue74(N4-05)	Vietnam	0	0	0	0	0	0.999	0	0	Sub-pop 6
H18314	越 75(N3-05)	Yue75(N3-05)	Vietnam	0	0	0	0	0	0.999	0	0	Sub-pop 6
H18315	越 76(7133)	Yue76(7133)	Vietnam	0	0	0	0	0	0.999	0	0	Sub-pop 6
H18316	越 77(NEP 98)	Yue77(NEP 98)	Vietnam	0	0	0	0	0	0.999	0	0	Sub-pop 6
H18317	越 78(NEP QUYT)	Yue78(NEP QUYT)	Vietnam	0	0	0	0	0	0.999	0	0	Sub-pop 6
H18318	越 79(DB1)	Yue79(DB1)	Vietnam	0	0	0	0	0	0.999	0	0	Sub-pop 6
H18319	越 80(DB1)	Yue80(DB1)	Vietnam	0	0	0	0	0	0.999	0	0	Sub-pop 6
H18320	越 81(61)	Yue81(61)	Vietnam	0	0	0	0	0	0.999	0	0	Sub-pop 6
H18321	越 82(17.11-2)	Yue82(17.11-2)	Vietnam	0	0.001	0.002	0	0.011	0.984	0	0.001	Sub-pop 6
H18322	越 83(BM207)	Yue83(BM207)	Vietnam	0	0	0	0	0	0.999	0	0	Sub-pop 6
H18323	越 84(NX30)	Yue84(NX30)	Vietnam	0.001	0.005	0.001	0	0.004	0.988	0.001	0	Sub-pop 6
H18324	越 85(HT9)	Yue85(HT9)	Vietnam	0	0	0	0	0	0.999	0	0	Sub-pop 6
H18325	越 86(KHAU CUI)	Yue86(KHAU CUI)	Vietnam	0	0	0	0	0	0.999	0	0	Sub-pop 6
H18326	越 87(69)	Yue87(69)	Vietnam	0	0	0	0	0	0.999	0	0	Sub-pop 6

H18327	越 88(60)	Yue88(60)	Vietnam	0	0	0	0	0	0.999	0	0	Sub-pop 6
H18328	越 89(54)	Yue89(54)	Vietnam	0	0	0	0	0	0.999	0	0	Sub-pop 6
H18329	越 90(AC5-T)	Yue90(AC5-T)	Vietnam	0	0	0	0	0	0.999	0	0	Sub-pop 6
H18330	越 91(N16-12)	Yue91(N16-12)	Vietnam	0	0	0	0	0	0.999	0	0	Sub-pop 6
H18331	越 93(N2-08-1)	Yue93(N2-08-1)	Vietnam	0	0	0	0	0	0.999	0	0	Sub-pop 6
H18332	越 9412.6-1	Yue9412.6-1	Vietnam	0	0	0	0	0	0.999	0	0	Sub-pop 6
H18333	越 95(P11)	Yue95(P11)	Vietnam	0	0	0	0	0	0.999	0	0	Sub-pop 6
H18334	越 96（688）	Yue96（688）	Vietnam	0	0.002	0.003	0	0.001	0.987	0.006	0	Sub-pop 6
H18335	越 97（12.6）	Yue97（12.6）	Vietnam	0	0	0	0	0	0.999	0	0	Sub-pop 6
H18336	越 98（49.1）	Yue98（49.1）	Vietnam	0	0	0	0	0	0.999	0	0	Sub-pop 6
H18337	越 99（8.3-1-22）	Yue99（8.3-1-22）	Vietnam	0	0	0	0	0	0.999	0	0	Sub-pop 6
H18338	越 100（BM205）	Yue100（BM205）	Vietnam	0	0	0	0	0	0.999	0	0	Sub-pop 6
H18339	越 101（NEP 87）	Yue101（NEP 87）	Vietnam	0	0	0	0	0	0.999	0	0	Sub-pop 6
H18340	越 102（BM216）	Yue102（BM216）	Vietnam	0	0	0	0.412	0	0.588	0	0	Admix
H18341	越 103（1）	Yue103（1）	Vietnam	0	0	0	0	0	0.999	0	0	Sub-pop 6
H18342	越 104（LHD5）	Yue104（LHD5）	Vietnam	0	0.001	0.007	0	0.002	0.988	0.001	0.001	Sub-pop 6
H18343	越 105（X26）	Yue105（X26）	Vietnam	0	0	0	0	0	0.999	0	0	Sub-pop 6
H18344	越 106（BM214）	Yue106（BM214）	Vietnam	0	0	0	0	0	0.999	0	0	Sub-pop 6
H18345	越 107（BM217）	Yue107（BM217）	Vietnam	0	0	0	0	0	0.999	0	0	Sub-pop 6
H18346	越 109（9603）	Yue109（9603）	Vietnam	0	0	0	0	0	0.999	0	0	Sub-pop 6

H18347	越 110（9）	Yue110（9）	Vietnam	0	0	0	0	0	0.999	0	0	Sub-pop 6
H18348	越 111（N98）	Yue111（N98）	Vietnam	0	0	0	0	0	0.999	0	0	Sub-pop 6
H18349	越 112（4）	Yue112（4）	Vietnam	0	0	0	0	0	0.999	0	0	Sub-pop 6
H18350	越 113（KD 中国）	Yue113（KDChina）	Vietnam	0	0	0	0	0	0.999	0	0	Sub-pop 6
H18351	越 114（HT6）	Yue114（HT6）	Vietnam	0	0	0	0	0	0.999	0	0	Sub-pop 6
H18352	越 115（5）	Yue115（5）	Vietnam	0	0.001	0.025	0	0	0.974	0	0	Sub-pop 6
H18353	越 116（8）	Yue116（8）	Vietnam	0	0	0	0	0	0.999	0	0	Sub-pop 6
H18354	越 117（HT9）	Yue117（HT9）	Vietnam	0	0	0	0	0	0.999	0	0	Sub-pop 6
H18355	越 118（2）	Yue118（2）	Vietnam	0	0	0	0	0	0.999	0	0	Sub-pop 6
H18356	越 119（N02）	Yue119（N02）	Vietnam	0	0	0	0	0	0.999	0	0	Sub-pop 6
H18357	越 120（3）	Yue120（3）	Vietnam	0	0	0	0	0	0.999	0	0	Sub-pop 6
H18358	越 121（1）	Yue121（1）	Vietnam	0	0	0	0	0	0.999	0	0	Sub-pop 6
H18359	飞来凤	Feilaifeng	Wuxi, Jiangsu	0	0	0	0	0	0.999	0	0	Sub-pop 6
H18360	南农粳 3786	Nannongjing3786	Nanjing, Jiangsu	0	0	0	0	0	0.999	0	0	Sub-pop 6
H18361	24248	24248	Nanjing, Jiangsu	0	0	0	0	0	0.999	0	0	Sub-pop 6
H18362	南农粳 4004	Nannongjing4004	Nanjing, Jiangsu	0	0	0	0	0	0.999	0	0	Sub-pop 6
H18363	南农粳 4016	Nannongjing4016	Nanjing, Jiangsu	0	0	0	0	0	0.999	0	0	Sub-pop 6
H18364	紫尖武粳	Zijianwujing	Nanjing, Jiangsu	0	0.007	0.006	0	0.001	0.984	0.001	0	Sub-pop 6
H18365	武香 99-8	Wuxiang99-8	Wujin, Jiangsu	0	0	0	0	0	0.999	0	0	Sub-pop 6
H18366	武运粳 8 号	Wuyujing8	Wujin, Jiangsu	0	0	0	0	0	0.999	0	0	Sub-pop 6

H18367	南农粳 002	Nannongjing002	Nanjing, Jiangsu	0	0	0	0	0	0.999	0	0	Sub-pop 6
H18368	南农粳 004	Nannongjing004	Nanjing, Jiangsu	0	0	0	0	0	0.999	0	0	Sub-pop 6
H18369	淮稻 5 号	Huaidao5hao	Huaian, Jiangsu	0	0	0	0	0.532	0.467	0	0	Admix
H18370	中作 93	Zhongzuo93	Tongzhou, Beijing	0	0	0.002	0.001	0.577	0.42	0	0	Admix
H18371	盐稻 9 号	Yandao9	Yancheng, Jiangsu	0	0	0	0	0.577	0.422	0	0	Admix
H18372	连粳 4 号	Lianjing4	Lianyungang, Jiangsu	0	0	0	0	0.592	0.407	0	0	Admix
H18373	津稻 1007	Jindao1007	Dongli, Tianjin	0	0	0	0	0.609	0.39	0	0	Admix
H18374	华粳 5 号	Huajing5	Huaibei, Jiangsu	0	0	0.003	0	0.716	0.279	0	0	Admix
H18375	华粳 6 号	Huajing6	Huaibei, Jiangsu	0	0	0.001	0	0.622	0.375	0	0	Admix
H18376	扬辐粳 7 号	Yangfujing7	Lixiahe, Jiangsu	0	0	0.001	0	0.642	0.356	0	0	Admix
H18377	扬辐粳 8 号	Yangfujing8	Lixiahe, Jiangsu	0	0	0	0	0.999	0	0	0	Sub-pop 5
H18378	镇稻 99	Zhendao99	Zhenjiang, Jiangsu	0	0	0	0	0.999	0	0	0	Sub-pop 5
H18379	南粳 42	Nanjing42	Nanjing, Jiangsu	0	0	0	0	0.999	0	0	0	Sub-pop 5
H18380	连粳 9823	Lianjing9823	Lianyungang, Jiangsu	0	0	0	0	0.999	0	0	0	Sub-pop 5
H18381	辉丰 1	Huifeng1	Yancheng, Jiangsu	0	0	0	0	0.999	0	0	0	Sub-pop 5
H18382	辉丰 2	Huifeng2	Yancheng, Jiangsu	0	0	0	0	0.999	0	0	0	Sub-pop 5
H18383	盐稻 8 号	Yandao8	Yancheng, Jiangsu	0	0	0	0	0.999	0	0	0	Sub-pop 5
H18384	武运粳 21 号	Wuyujing21	Wujin, Jiangsu	0	0	0	0	0.999	0	0	0	Sub-pop 5

H18385	莎莎妮	Shashani	Haerbin, Heilongjiang	0	0	0	0	0.999	0	0	0	Sub-pop 5
H18386	牡粘 4 号	Muzhan4	Mudanjiang, Heilongjiang	0	0	0	0	0.999	0	0	0	Sub-pop 5
H18387	牡丹江 29	Mudanjiang29	Mudanjiang, Heilongjiang	0	0	0	0	0.999	0	0	0	Sub-pop 5
H18388	牡丹江 28	Mudanjiang28	Mudanjiang, Heilongjiang	0	0	0	0	0.999	0	0	0	Sub-pop 5
H18389	牡丹江 27	Mudanjiang27	Mudanjiang, Heilongjiang	0	0	0	0	0.999	0	0	0	Sub-pop 5
H18390	垦粘 2 号	Kenzhan2	Nongken, Heilongjiang	0	0	0	0	0.999	0	0	0	Sub-pop 5
H18391	黑粳 8 号	Heijing8	Haerbin, Heilongjiang	0	0	0	0	0.999	0	0	0	Sub-pop 5
H18392	合粳 1 号	Hejing1	Haerbin, Heilongjiang	0	0	0	0	0.999	0	0	0	Sub-pop 5
H18393	北稻 4 号	Beidao4	Haerbin, Heilongjiang	0	0	0	0	0.999	0	0	0	Sub-pop 5
H18394	北稻 3 号	Beidao3	Haerbin, Heilongjiang	0	0	0	0	0.999	0	0	0	Sub-pop 5
H18395	绥粳 12 号	Suijing12	Suihua, Heilongjiang	0	0	0	0	0.999	0	0	0	Sub-pop 5
H18396	松粳 12	Songjing12	Songhuajiang, Heilongjiang	0	0	0	0	0.999	0	0	0	Sub-pop 5

H18397	松粳 11	Songjing11	Songhuajiang, Heilongjiang	0	0	0	0	0.999	0	0	0	Sub-pop 5
H18398	松粳 10	Songjing10	Songhuajiang, Heilongjiang	0	0	0	0	0.999	0	0	0	Sub-pop 5
H18399	东农 430	Dongnong430	Haerbin, Heilongjiang	0	0	0	0	0.999	0	0	0	Sub-pop 5
H18400	东农 424	Dongnong424	Haerbin, Heilongjiang	0	0	0	0	0.999	0	0	0	Sub-pop 5
H18401	龙糯 3 号	Longnuo3	Haerbin, Heilongjiang	0	0	0	0	1	0	0	0	Sub-pop 5
H18402	龙粳 28	Longjing28	Haerbin, Heilongjiang	0	0	0	0	0.999	0	0	0	Sub-pop 5
H18403	龙粳 27	Longjing27	Haerbin, Heilongjiang	0	0	0	0	0.999	0	0	0	Sub-pop 5
H18404	龙粳 26	Longjing26	Haerbin, Heilongjiang	0	0	0	0	0.999	0	0	0	Sub-pop 5
H18405	龙粳 25	Longjing25	Haerbin, Heilongjiang	0	0	0	0	0.999	0	0	0	Sub-pop 5
H18406	龙粳 24	Longjing24	Haerbin, Heilongjiang	0	0	0	0	0.999	0	0	0	Sub-pop 5
H18407	龙粳 22	Longjing22	Haerbin, Heilongjiang	0	0	0	0	0.999	0	0	0	Sub-pop 5
H18408	龙粳 21	Longjing21	Haerbin, Heilongjiang	0	0	0	0	0.999	0	0	0	Sub-pop 5

H18409	龙粳 20	Longjing20	Haerbin, Heilongjiang	0	0	0	0	0.999	0	0	0	Sub-pop 5
H18410	龙粳 19	Longjing19	Haerbin, Heilongjiang	0	0	0	0	0.999	0	0	0	Sub-pop 5
H18411	龙粳 18	Longjing18	Haerbin, Heilongjiang	0	0	0	0	0.999	0	0	0	Sub-pop 5
H18412	龙粳 17	Longjing17	Haerbin, Heilongjiang	0	0	0	0	0.999	0	0	0	Sub-pop 5
H18413	龙粳 16	Longjing16	Haerbin, Heilongjiang	0	0	0	0	0.999	0	0	0	Sub-pop 5
H18414	龙粳 15	Longjing15	Haerbin, Heilongjiang	0	0	0	0	0.999	0	0	0	Sub-pop 5
H18415	中龙稻 1 号	Zhonglongdao1	Haerbin, Heilongjiang	0	0	0	0	0.999	0	0	0	Sub-pop 5
H18416	龙稻 8 号	Zongdao8	Haerbin, Heilongjiang	0	0	0	0	0.999	0	0	0	Sub-pop 5
H18417	龙稻 6 号	Longdao6	Haerbin, Heilongjiang	0	0	0	0	0.999	0	0	0	Sub-pop 5
H18418	龙稻 5 号	Longdao5	Haerbin, Heilongjiang	0	0	0	0	0.999	0	0	0	Sub-pop 5
H18419	龙稻 4 号	Longdao4	Haerbin, Heilongjiang	0	0	0	0	0.999	0	0	0	Sub-pop 5
H18420	垦稻 19	Kendao19	Haerbin, Heilongjiang	0	0	0	0	0.999	0	0	0	Sub-pop 5

H18421	垦稻 18	Kendao18	Haerbin, Heilongjiang	0	0	0	0	0.999	0	0	0	Sub-pop 5
H18422	垦稻 12	Kendao12	Haerbin, Heilongjiang	0	0	0	0	0.999	0	0	0	Sub-pop 5
H18423	垦稻 13	Kendao13	Haerbin, Heilongjiang	0	0	0	0	0.999	0	0	0	Sub-pop 5
H18424	垦稻 20	Kendao20	Haerbin, Heilongjiang	0	0	0	0	0.999	0	0	0	Sub-pop 5
H18425	龙盾 106	Longdun106	Haerbin, Heilongjiang	0	0	0	0	0.999	0	0	0	Sub-pop 5
H18426	龙盾 105	Longdun105	Haerbin, Heilongjiang	0	0	0	0	0.999	0	0	0	Sub-pop 5
H18427	三江 2 号	Sanjiang2	Haerbin, Heilongjiang	0	0	0	0	0.999	0	0	0	Sub-pop 5
H18428	农香 21	Nongxiang21	Changsha, Hunan	0	0	0	0	0.999	0	0	0	Sub-pop 5
H18429	农香 25	Nongxiang25	Changsha, Hunan	0	0	0	0	0.999	0	0	0	Sub-pop 5
H18430	农香 26	Nongxiang26	Changsha, Hunan	0.001	0.001	0.002	0	0.985	0.009	0.001	0.001	Sub-pop 5
H18431	玉针香	Yuzhenxiang	Changsha, Hunan	0	0	0	0	0.999	0	0	0	Sub-pop 5
H18432	黄华占	Huanghuazhan	Changsha, Hunan	0	0	0	0	0.999	0	0	0	Sub-pop 5
H18433	丰优晚 8 号	Fengyouwan8	Changsha, Hunan	0	0	0	0	0.999	0	0	0	Sub-pop 5
H18434	日本晴	Ribenqing	Haerbin, Heilongjiang	0	0	0	0	0.999	0	0	0	Sub-pop 5

H18435	屉锦	Tijin	Haerbin, Heilongjiang	0	0	0	0	0.999	0	0	0	Sub-pop 5
H18436	M1004	M1004	Haerbin, Heilongjiang	0	0	0	0	0.999	0	0	0	Sub-pop 5
H18437	中国 91	Zhongguo91	Haerbin, Heilongjiang	0	0	0	0	0.999	0	0	0	Sub-pop 5
H18438	秋田小町	Qiutainxiaoding	Haerbin, Heilongjiang	0	0	0	0	0.999	0	0	0	Sub-pop 5
H18439	彩	Cai	Haerbin, Heilongjiang	0	0	0	0	0.999	0	0	0	Sub-pop 5
H18440	秋光	Qiuguang	Haerbin, Heilongjiang	0.001	0.002	0.002	0	0.978	0.015	0.001	0.002	Sub-pop 5
H18441	优质 Yue 光	Youzhiyueguang	Haerbin, Heilongjiang	0	0	0	0	0.999	0	0	0	Sub-pop 5
H18442	Kasalath	Kasala	Haerbin, Heilongjiang	0	0	0	0	0.999	0	0	0	Sub-pop 5
H18443	香川无心白米（オオセト）	Xiangchuanwuxinbaimi	Haerbin, Heilongjiang	0	0	0	0	0.999	0	0	0	Sub-pop 5
H18444	董铮 1640	Dongzheng1640	Hongze, Jiangsu	0	0	0	0	0.999	0	0	0	Sub-pop 5
H18445	董铮武运粳 21	Dongzhengwuyujing21	Hongze, Jiangsu	0.999	0	0	0	0	0	0	0	Sub-pop 1
H18446	黑米籼稻	Heimixiandao	Nanjing, Jiangsu	0.999	0	0	0	0	0	0	0	Sub-pop 1
H18447	黑米粳稻	Heimijingdao	Nanjing, Jiangsu	0.999	0	0	0	0	0	0	0	Sub-pop 1
H18448	紫稻	Zidao	Nanjing, Jiangsu	0.999	0	0	0	0	0	0	0	Sub-pop 1

H18449	9311	9311	Yangzhou, Jiangsu	0.999	0	0	0	0	0	0	0	Sub-pop 1
H18450	杂草稻	Zacaodao	Nanjing, Jiangsu	0.999	0	0	0	0	0	0	0	Sub-pop 1
H18451	有芒 429bp	Youmang429	Nanjing, Jiangsu	0.999	0	0	0	0	0	0	0	Sub-pop 1
H18452	弯曲 429bp	Wanqu429	Nanjing, Jiangsu	1	0	0	0	0	0	0	0	Sub-pop 1
H18453	直立 429bp	Zhili429	Nanjing, Jiangsu	0.999	0	0	0	0	0	0	0	Sub-pop 1
H18454	泗好 4029	Si4029	Sihong, Jiangsu	1	0	0	0	0	0	0	0	Sub-pop 1
H18455	泗好 4031	Si4031	Sihong, Jiangsu	0.999	0	0	0	0	0	0	0	Sub-pop 1
H18456	泗好 4033	Si4033	Sihong, Jiangsu	1	0	0	0	0	0	0	0	Sub-pop 1
H18457	泗好 4039	Si4039	Sihong, Jiangsu	1	0	0	0	0	0	0	0	Sub-pop 1
H18458	泗好 4040	Si4040	Sihong, Jiangsu	1	0	0	0	0	0	0	0	Sub-pop 1
H18459	泗好 4041	Si4041	Sihong, Jiangsu	1	0	0	0	0	0	0	0	Sub-pop 1
H18460	泗好 4049	Si4049	Sihong, Jiangsu	1	0	0	0	0	0	0	0	Sub-pop 1
H18461	泗好 4079	Si4079	Sihong, Jiangsu	0.999	0	0	0	0	0	0	0	Sub-pop 1
H18462	泗好 4081ck	Si4081	Sihong, Jiangsu	0.999	0	0	0	0	0	0	0	Sub-pop 1
H18463	泗好 4082ck	Si4082	Sihong, Jiangsu	1	0	0	0	0	0	0	0	Sub-pop 1
H18464	泗好 4139	Si4139	Sihong, Jiangsu	1	0	0	0	0	0	0	0	Sub-pop 1
H18465	泗好 4152	Si4152	Sihong, Jiangsu	0.999	0	0	0	0	0	0	0	Sub-pop 1
H18466	泗好 4161	Si4161	Sihong, Jiangsu	1	0	0	0	0	0	0	0	Sub-pop 1
H18467	泗好 4229	Si4229	Sihong, Jiangsu	1	0	0	0	0	0	0	0	Sub-pop 1
H18468	泗好 4230	Si4230	Sihong, Jiangsu	1	0	0	0	0	0	0	0	Sub-pop 1

H18469	泗好 4251	Si4251	Sihong, Jiangsu	1	0	0	0	0	0	0	0	Sub-pop 1
H18470	泗好 4252	Si4252	Sihong, Jiangsu	1	0	0	0	0	0	0	0	Sub-pop 1
H18471	泗好 4259	Si4259	Sihong, Jiangsu	1	0	0	0	0	0	0	0	Sub-pop 1
H18472	泗好 4263	Si4263	Sihong, Jiangsu	1	0	0	0	0	0	0	0	Sub-pop 1
H18473	泗好 4280	Si4280	Sihong, Jiangsu	1	0	0	0	0	0	0	0	Sub-pop 1
H18474	泗好 4330	Si4330	Sihong, Jiangsu	1	0	0	0	0	0	0	0	Sub-pop 1
H18475	泗好 4360	Si4360	Sihong, Jiangsu	1	0	0	0	0	0	0	0	Sub-pop 1
H18476	泗好 4364	Si4364	Sihong, Jiangsu	1	0	0	0	0	0	0	0	Sub-pop 1
H18477	低 glu 稻(糖尿病专用)	Digudao	Sihong, Jiangsu	1	0	0	0	0	0	0	0	Sub-pop 1
H18478	泗好 4386（银香玉）	Si4386	Sihong, Jiangsu	1	0	0	0	0	0	0	0	Sub-pop 1
H18479	泗好 4141	Sihao4141	Sihong, Jiangsu	1	0	0	0	0	0	0	0	Sub-pop 1
H18480	宁粳恢 096	Ningjinghui096	Nanjing, Jiangsu	1	0	0	0	0	0	0	0	Sub-pop 1
H18481	宁粳恢 117	Ningjinghui117	Nanjing, Jiangsu	1	0	0	0	0	0	0	0	Sub-pop 1
H18482	宁粳恢 145	Ningjinghui145	Nanjing, Jiangsu	1	0	0	0	0	0	0	0	Sub-pop 1
H18483	宁粳恢 166	Ningjinghui166	Nanjing, Jiangsu	1	0	0	0	0	0	0	0	Sub-pop 1
H18484	宁粳恢 208	Ningjinghui208	Nanjing, Jiangsu	1	0	0	0	0	0	0	0	Sub-pop 1
H18485	宁粳恢 210	Ningjinghui210	Nanjing, Jiangsu	1	0	0	0	0	0	0	0	Sub-pop 1
H18486	宁粳恢 237	Ningjinghui237	Nanjing, Jiangsu	1	0	0	0	0	0	0	0	Sub-pop 1
H18487	宁粳恢 246	Ningjinghui246	Nanjing, Jiangsu	1	0	0	0	0	0	0	0	Sub-pop 1

H18488	宁粳恢 260	Ningjinghui260	Nanjing, Jiangsu	1	0	0	0	0	0	0	0	Sub-pop 1
H18489	宁粳恢 285	Ningjinghui285	Nanjing, Jiangsu	1	0	0	0	0	0	0	0	Sub-pop 1
H18490	宁粳恢 286	Ningjinghui286	Nanjing, Jiangsu	1	0	0	0	0	0	0	0	Sub-pop 1
H18491	宁粳恢 290	Ningjinghui290	Nanjing, Jiangsu	1	0	0	0	0	0	0	0	Sub-pop 1
H18492	宁粳恢 292	Ningjinghui292	Nanjing, Jiangsu	0.999	0	0	0	0	0	0	0	Sub-pop 1
H18493	宁粳恢 293	Ningjinghui293	Nanjing, Jiangsu	0.999	0	0	0	0	0	0	0	Sub-pop 1
H18494	宁粳恢 296	Ningjinghui296	Nanjing, Jiangsu	0.999	0	0	0	0	0	0	0	Sub-pop 1
H18495	宁粳恢 298	Ningjinghui298	Nanjing, Jiangsu	0.999	0	0	0	0	0	0	0	Sub-pop 1
H18496	宁粳恢 338	Ningjinghui338	Nanjing, Jiangsu	0.999	0	0	0	0	0	0	0	Sub-pop 1
H18497	洪引 1009	Hongyin1009	Nanjing, Jiangsu	0.999	0	0	0	0	0	0	0	Sub-pop 1
H18498	洪引 1010	Hongyin1010	Nanjing, Jiangsu	0.999	0	0	0	0	0	0	0	Sub-pop 1
H18499	洪引 1011	Hongyin1011	Nanjing, Jiangsu	0.999	0	0	0	0	0	0	0	Sub-pop 1
H18500	洪引 1012	Hongyin1012	Nanjing, Jiangsu	0.999	0	0	0	0	0	0	0	Sub-pop 1
H18501	彩	Cai1	Haerbin, Heilongjiang	0.999	0	0	0	0	0	0	0	Sub-pop 1
H18502	南农粳 1R	Nannongjing1R	Nanjing, Jiangsu	0.999	0	0	0	0	0	0	0	Sub-pop 1
H18503	南农粳 2R	Nannongjing2R	Nanjing, Jiangsu	0.976	0	0.001	0	0	0.021	0.002	0	Sub-pop 1
H18504	南农粳 3R	Nannongjing3R	Nanjing, Jiangsu	0.992	0	0.001	0.001	0.001	0.002	0.001	0.001	Sub-pop 1

Table S3. Analysis of molecular variance (AMOVA) for the 8 subpopulations of rice varieties.

Source of variation	df	Sum of squares	Variance components	Percentage of variation	P Value
Among populations	8	20750.989	49.85622 Va	48.21	P<0.01
Within populations	466	24960.277	53.56283 Vb	51.79	P<0.01
Total	474	45711.266	103.41905		

Table S4. Pairwise population differentiation according to groups of populations as measured by F_{st} values using Arlequin software ver. 3.5 .

Subpopulation	Sub-pop 1	Sub-pop 2	Sub-pop 3	Sub-pop 4	Sub-pop 5	Sub-pop 6	Sub-pop 7	Sub-pop 8
Sub-pop 1	-	0.71	0.66	0.75	0.65	0.69	0.67	0.68
Sub-pop 2	0.52	-	0.48	0.68	0.57	0.64	0.58	0.63
Sub-pop 3	0.45	0.44	-	0.58	0.55	0.54	0.56	0.60
Sub-pop 4	0.47	0.57	0.45	-	0.66	0.57	0.70	0.70
Sub-pop 5	0.44	0.48	0.41	0.48	-	0.62	0.59	0.52
Sub-pop 6	0.47	0.52	0.42	0.45	0.45	-	0.65	0.65
Sub-pop 7	0.51	0.59	0.48	0.59	0.50	0.53	-	0.53
Sub-pop 8	0.48	0.59	0.48	0.55	0.44	0.51	0.56	-

Nei's genetic distances appear above the diagonal and Pairwise Fst values appear below the diagonal. All *Fst* values are significant (P<0.01).

Table S5. Comparison of D' values of LD for pairwise loci in each subpopulation.

Sub populations	Number of significant LD locus pairs [a]	Number of significant LD locus pairs/Total number of locus pairs (%) [b]	Frequency of D' value (P<0.05)					Mean of D′ [c]
			0-0.2	0.21-0.4	0.41-0.6	0.61-0.8	0.81-1.0	
Sub-pop 1	1723	8.21	0	185	602	530	405	0.653
Sub-pop 2	439	3.74	0	23	131	141	144	0.705
Sub-pop 3	1425	8.63	5	149	323	451	497	0.696
Sub-pop 4	1180	8.06	0	28	176	280	696	0.814
Sub-pop 5	1434	8.83	1	120	307	337	669	0.742
Sub-pop 6	1215	8.4	15	256	307	274	363	0.639
Sub-pop 7	409	3.08	1	50	135	90	133	0.675
Sub-pop 8	1728	8.25	0	189	601	533	405	0.653
	9553							

[a] LD means linkage disequilibrium.

[b] Ratio between the number of significant LD locus pairs and total number of locus pairs.

[c] D′ means standardized disequilibrium coefficients.

Table S6. Marker–trait associations with *P* value <0.05, their equivalent false discovery rate probability (FDR), proportion of phenotypic variance explained (PVE), marker position on chromosome derived from 262 markers and 474 rice accessions for PH.

Traits	Year	SSR Marker	Chromosome	Position (cM) [a]	Position (bp) [b]		P value	PVE (%)	FDR
					Start pos	End pos			
PH	2018	RM1240	11	6.5	1660893	1660932	3.61E-05	10.31	3.13E-03
		RM324	2	51.1	11388913	11388939	1.75E-02	2.93	1.88E-02
		RM6215	8	66.8	19058582	19058608	1.85E-02	4.9	2.19E-02
		RM249	5	50.2	10776494	10776621	1.95E-02	3.9	2.50E-02
		RM161	5	96.9			2.10E-02	6.92	3.13E-02
		RM3330	6	61.6	10907997	10908028	2.94E-02	5.48	3.44E-02
		RM265	1	153.5	35196573	35196681	3.57E-02	3.53	3.75E-02
		RM3589	7	89.8	25054610	25054631	3.71E-02	5.01	4.06E-02
		RM7309	6	100.3	25914707	25914734	4.08E-02	5.51	4.38E-02
		RM454	6	99.3			4.50E-02	2.07	4.69E-02
	2019	RM524	9	42.5	12871621	12871642	5.11E-03	6.41	1.18E-02
		RM234	7	93.9	25420132	25420157	5.00E-02	5.06	5.00E-02

[a] The estimated map position (cM) and [b] physical position (bp) was inferred the Gremene ("http://www.gramene.org/markers") and NCBI ("http://blast.ncbinlm.nih.gov/Blast.cgi").

Table S7. Marker–trait associations with *P* value <0.05, their equivalent false discovery rate probability (FDR), proportion of phenotypic variance explained (PVE), marker position on chromosome derived from 262 markers and 474 rice accessions for PL.

Traits	Year	SSR Marker	Chromosome	Position (cM) [a]	Position (bp) [b]		P value	PVE (%)	FDR
					Start pos	End pos			
PL	2018	RM8243	8	50.8	8930116	8930137	6.11E-03	5.63	1.18E-02
		RM24481	9	63	17210236	17210327	9.18E-03	7.88	1.76E-02
		RM249	5	50.2	10776494	10776621	1.42E-02	4.1	2.65E-02
		RM7598	2	126.4	29846409	29846432	2.76E-02	3.37	3.24E-02
		RM598	5	62.7	16676126	16676152	3.03E-02	2.98	3.53E-02
		RM434	9	57.7	15609041	15609064	3.12E-02	3.93	3.82E-02
		RM3331	12	89.5	23528087	23528116	3.23E-02	3.59	4.41E-02
		RM410	9	79.3	514907	514934	3.43E-02	3.87	5.00E-02
	2019	RM7545	10	7.6	3785115	3785186	9.12E-03	9.67	2.06E-02
		RM1108	10	55.3	18716363	18716386	1.59E-02	3.34	2.35E-02
		RM1231	1	98.5	29472375	29472404	2.25E-02	6.28	2.65E-02
		RM159	5	5.4	488027	488281	3.15E-02	5.43	4.12E-02
		RM512	12	39.4	5104402	5104421	3.44E-02	2.57	4.41E-02
		RM171	10	73	19048795	19049123	3.96E-02	2.14	4.71E-02
		RM25	8	52.2			4.55E-02	4.57	5.00E-02

[a] The estimated map position (cM) and [b] physical position (bp) was inferred the Gremene ("http://www.gramene.org/markers") and NCBI ("http://blast.ncbinlm.nih.gov/Blast.cgi").

Table S8. Marker–trait associations with *P* value <0.05, their equivalent false discovery rate probability (FDR), proportion of phenotypic variance explained (PVE), marker position on chromosome derived from 262 markers and 474 rice accessions for 1IN.

Traits	Year	SSR Marker	Chromosome	Position (cM) [a]	Position (bp) [b]		P value	PVE (%)	FDR
					Start pos	End pos			
1IN	2018	RM471	4	53.8	19007714	19007737	1.54E-02	4.38	1.67E-02
		RM6976	8	92.2	23551198	23551242	1.55E-02	7.45	2.50E-02
		RM437	5	31.5	3815948	3815973	4.83E-02	1.29	5.00E-02
	2019	RM348	4	160.8	32869438	32869458	8.89E-04	4.47	1.00E-02
		RM122	5	3	279748	279775	2.73E-03	3.9	1.50E-02
		RM4835	4	18.3	19135687	19135792	6.25E-03	4.93	2.00E-02
		RM72	8	60.9			1.69E-02	5.89	2.50E-02
		RM512	12	39.4	5104402	5104421	2.29E-02	2.78	4.00E-02
		RM5746	12	39.4	5092032	5092055	2.48E-02	6.19	4.50E-02
		RM171	10	73	23072213	23072266	4.10E-02	2.12	5.00E-02

[a] The estimated map position (cM) and [b] physical position (bp) was inferred the Gremene (“http://www.gramene.org/markers”) and NCBI (“http://blast.ncbinlm.nih.gov/Blast.cgi”).

Table S9. Marker–trait associations with *P* value <0.05, their equivalent false discovery rate probability (FDR), proportion of phenotypic variance explained (PVE), marker position on chromosome derived from 262 markers and 474 rice accessions for 2IN.

Traits	Year	SSR Marker	Chromosome	Position (cM) [a]	Position (bp) [b]		P value	PVE (%)	FDR
					Start pos	End pos			
2IN	2018	RM264	8	138.2			8.97E-03	5.71	2.73E-02
		RM5479	12	95.4	24446205	24446246	1.20E-02	6.75	3.64E-02
		RM345	6	123.9	30864845	30864999	3.30E-02	2.93	4.09E-02
		RM218	3	67.8	8375236	8375283	3.50E-02	3.85	4.55E-02
		RM1-003	1	194			3.50E-02	4.46	5.00E-02
	2019	RM450	2	122.8	28652656	28652689	1.44E-02	3.75	2.27E-02
		RM161	5	96.9			2.48E-02	6.78	3.64E-02
		RM583	1	38.8	8328958	8329017	2.83E-02	3.02	4.09E-02
		RM244	10	15			2.85E-02	3.01	4.55E-02
		RM1108	10	55.3	18716363	18716386	4.82E-02	2.71	5.00E-02

[a] The estimated map position (cM) and [b] physical position (bp) was inferred the Gremene ("http://www.gramene.org/markers") and NCBI ("http://blast.ncbinlm.nih.gov/Blast.cgi").

Table S10. Marker–trait associations with *P* value <0.05, their equivalent false discovery rate probability (FDR), proportion of phenotypic variance explained (PVE), marker position on chromosome derived from 262 markers and 474 rice accessions for 3IN.

Traits	Year	SSR Marker	Chromosome	Position (cM) [a]	Position (bp) [b]		P value	PVE (%)	FDR
					Start pos	End pos			
3IN	2018	RM7345	3	48.8	10958945	10958968	1.47E-03	5.04	7.14E-03
		RM11	7	93.8	19204350	19204379	9.26E-03	4.36	2.14E-02
		RM1108	10	55.3	18716363	18716386	1.99E-02	3.22	3.21E-02
		RM463	12	75.5	22159508	22159527	2.04E-02	3.55	3.57E-02
		RM598	5	62.7	16676126	16676152	2.33E-02	3.13	3.93E-02
		RM6863	8	16.4	2005990	2006016	3.99E-02	4.37	5.00E-02
	2019	RM1313	2	51.1	11262096	11262133	1.83E-02	4.59	2.31E-02
		RM5479	12	95.4	24446205	24446246	1.85E-02	6.43	2.69E-02
		RM258	10	48.8	17570591	17570612	2.42E-02	3.77	3.08E-02
		RM168	3	122.8	27898585	27898604	4.19E-02	4.33	4.62E-02

[a] The estimated map position (cM) and [b] physical position (bp) was inferred the Gremene ("http://www.gramene.org/markers") and NCBI ("http://blast.ncbinlm.nih.gov/Blast.cgi").

Table S11. Marker–trait associations with *P* value <0.05, their equivalent false discovery rate probability (FDR), proportion of phenotypic variance explained (PVE), marker position on chromosome derived from 262 markers and 474 rice accessions for 4IN.

Traits	Year	SSR Marker	Chromosome	Position (cM) [a]	Position (bp) [b]		P value	PVE (%)	FDR
					Start pos	End pos			
4IN	2018	RM1246	12	65.3	19156149	19156178	7.51E-03	4.83	2.06E-02
		RM8239	6	91.9	24555002	24555027	1.33E-02	3.08	2.35E-02
		RM5	1	93	23968577	23968606	2.00E-02	4.21	2.94E-02
		RM324	2	51.1	11388913	11388939	2.28E-02	2.79	3.24E-02
		RM524	9	42.5	12871621	12871642	3.88E-02	4.98	4.12E-02
		RM6215	8	66.8	19058582	19058608	4.64E-02	4.26	1.98E-01
		RM7563	4	68.3	22848896	22848923	4.75E-02	3.34	1.59E-01
	2019	RM161	5	96.9			1.09E-02	7.44	1.67E-02
		RM5475	3	137.9	30376088	30376129	1.42E-02	6.93	2.00E-02
		RM5849	3	18.4	4237901	4237978	2.81E-02	5.81	3.67E-02
		RM188	5	95.3	21994619	21994639	3.14E-02	4.84	4.00E-02
		RM5479	12	95.4	24446205	24446246	0.043126	5.75	4.67E-02

[a] The estimated map position (cM) and [b] physical position (bp) was inferred the Gremene ("http://www.gramene.org/markers") and NCBI ("http://blast.ncbinlm.nih.gov/Blast.cgi").

Table S12. Phenotypic effect of top two negative favorable alleles for PH, 2IN, 3IN, 4IN and top two positive favorable alleles significantly associated with PL and 1IN and their carrier varieties.

Trait	Locus-alleles	Phenotypic effect value		Mean	P value and its significance of F test of ANOVA for each locus	Carrier variety
		2018	2019			
PH	RM301-145	-14.57	-15.41	-14.99	0.000151 ***	Huaidao9
	RM301-150	-24.62	-23.48	-24.05		Youmang429
	RM301-160	-8.70	-8.59	-8.65		Maoguangdao
	RM301-170	-21.81	-23.71	-22.76		Nongxiang26
	RM348-130	-33.05	-33.33	-33.19	0.000186 ***	Yue2
	RM348-155	-22.58	-22.65	-22.62		Cai1
	RM305-195	-38.06	-38.11	-38.08	0.0000000279 ****	Tiejingqing
	RM305-205	-41.16	-41.61	-41.39		Xiaomandao
	RM305-210	-51.71	-51.13	-51.42		Maoguangdao
	RM305-215	-37.09	-37.13	-37.11		Huaidao9
	RM305-270	-41.79	-41.29	-41.54		Yue9
	RM305-370	-43.43	-43.20	-43.31		Yue4
	RM264-150	-32.28	-33.76	-33.02	0.000000101 ****	Nannongjing2R
	RM264-155	-28.75	-28.09	-28.42		Wandao68
	RM264-165	-24.79	-23.36	-24.08		Sanjiang2
	RM264-170	-22.37	-21.25	-21.81		Nannongjing1R
	RM264-180	-3.51	-4.72	-4.12		Yue30
	RM264-185	-13.61	-13.79	-13.70		Huaidao9
	RM264-190	-23.29	-25.18	-24.24		Yue14
	RM512-115	-21.61	-23.01	-22.31	0.000871 ***	Fuxiang1
	RM512-130	-5.83	-3.75	-4.79		Yue4
	RM512-220	-24.72	-23.29	-24.00		Youmang429
PL	RM1-100	4.79	4.62	4.70	0.00449 **	Yue38
	RM1-360	2.42	2.98	2.70		Yue96
	RM1-80	5.61	5.28	5.45		Yue30
	RM583-180	3.41	3.08	3.24	ns	Yue96
	RM583-185	3.95	2.84	3.40		Yue10
	RM338-135	2.99	2.91	2.95	0.000161 ****	Yue96
	RM338-150	1.11	1.00	1.06		Sanjiang2
	RM338-160	5.68	5.39	5.53		Yue26
	RM338-175	5.27	5.51	5.39		Yue38
	RM122-160	6.79	6.13	6.46	0.000629 ***	Yue6
	RM122-180	4.75	4.16	4.46		Yue2
	RM122-235	1.91	1.51	1.71		Yue96
	RM122-270	2.88	2.75	2.81		Yue10

	RM3330-110	3.09	3.80	3.44		Yue63
	RM3330-120	3.39	3.51	3.45	0.00231 **	Yue38
	RM3330-135	7.20	7.69	7.44		Yue96
	RM5380-115	8.58	7.98	8.28		Yue85
	RM5380-120	1.44	1.14	1.29	0.000130 ***	Yangmiaozhong
	RM5380-130	2.09	2.59	2.34		Yue68
	RM5380-90	3.76	3.25	3.51		Fuxiang1
	RM3533-125	1.99	1.52	1.76		Yue96
	RM3533-190	5.50	5.58	5.54	0.0000231 ****	Yue38
	RM3533-220	9.56	9.31	9.44		Yue63
	RM3533-275	2.54	2.05	2.30		Muzhan4
	RM3600-125	2.30	2.71	2.50		Yue76
	RM3600-130	3.81	6.78	5.30		Yue38
	RM3600-140	4.58	4.76	4.67	0.0342 *	Yue46
	RM3600-170	3.58	3.22	3.40		Yue58
	RM3600-180	3.10	3.75	3.42		Yue96
	RM3600-85	1.44	1.10	1.27		Qiutiandaxiedao
	RM6160-160	5.27	5.07	5.17		Hongyin1009
	RM6160-170	3.52	3.29	3.41	0.00011 ***	Yue96
	RM6160-180	4.21	4.00	4.11		Yue30
	RM6160-200	2.12	2.22	2.17		Yue12
1IN	RM301-130	5.85	5.93	5.89		Qiyunuo10
	RM301-140	0.50	0.96	0.73	0.0000366 ****	Yue4
	RM301-155	4.99	4.80	4.89		Yue119
	RM301-180	3.69	3.80	3.75		Yue24
	RM264-120	10.58	10.25	10.42		Heizhong
	RM264-130	5.55	5.80	5.67		Qiyunuo10
	RM264-140	4.17	4.75	4.46	0.000000563 ****	Yilimang
	RM264-160	2.88	2.59	2.74		Yue36
	RM264-180	1.59	1.26	1.42		Yue119
	RM264-195	5.46	5.07	5.26		Erlibie
	RM257-125	9.41	9.84	9.62		Huangkewanguangtou
	RM257-130	3.05	3.26	3.15		Qiyunuo10
	RM257-140	0.36	0.14	0.25	0.0000000323 ****	Yue36
	RM257-165	1.82	1.81	1.81		Zacaodao
	RM257-180	5.55	5.81	5.68		Yue4
	RM257-190	6.52	6.29	6.40		Zhonghua3
2IN	RM301-145	-1.04	-1.19	-1.12		Yandao6
	RM301-150	-1.10	-1.65	-1.37	ns	Si4364
	RM301-160	-2.08	-2.48	-2.28		Kendao12
	RM301-170	-1.53	-1.89	-1.71		Zhendao10

	RM5427-115	-1.45	-1.40	-1.42		Hongyin1010
	RM5427-120	-0.60	-0.71	-0.65		Yangmiaozhong
	RM5427-125	-2.68	-3.52	-3.10		Digudao
	RM5427-135	-0.46	-0.89	-0.68	0.00037 ***	Si4364
	RM5427-140	-1.89	-2.20	-2.05		Hongyin1012
	RM5427-150	-0.84	-0.30	-0.57		Kendao12
	RM5427-175	-1.13	-1.28	-1.20		Zhonghua3
	RM5427-195	-0.83	-0.31	-0.57		Yue11
	RM348-130	-7.64	-7.32	-7.48		Zhonghua3
	RM348-135	-0.33	-0.58	-0.46	0.0000675 ****	Yangmiaozhong
	RM348-155	-1.13	-1.31	-1.22		Si4364
	RM122-150	-3.25	-3.65	-3.45		Zhonghua3
	RM122-160	-1.48	-1.46	-1.47		Yue9
	RM122-180	-1.99	-1.58	-1.78	0.000156 ***	Yue3
	RM122-235	-0.43	-0.11	-0.27		Kendao12
	RM122-240	-0.30	-0.54	-0.42		Si4364
	RM305-195	-1.34	-1.61	-1.47		Shengdao808
	RM305-210	-1.09	-1.78	-1.44	ns	Kendao12
	RM305-370	-2.51	-2.43	-2.47		Aijiaoluganhuang
	RM512-115	-5.50	-5.60	-5.55		Zhonghua3
	RM512-130	-0.45	-0.76	-0.61	0.000492 ***	Yue3
	RM512-220	-1.11	-1.61	-1.36		Si4364
3IN	RM301-145	-2.95	-2.53	-2.74		Cbao
	RM301-150	-3.87	-3.57	-3.72	0.0085 **	Digudao
	RM301-160	-1.25	-1.48	-1.36		Longjing26
	RM301-170	-2.77	-3.52	-3.15		Sujing353
	RM348-130	-1.48	-1.18	-1.33		Zhonghua3
	RM348-135	-2.09	-2.03	-2.06	0.00793 **	Xudao3
	RM348-155	-3.16	-3.69	-3.42		Digudao
	RM122-160	-1.95	-1.30	-1.62	ns	Yue6
	RM122-180	-1.42	-1.40	-1.41		Yue1
	RM305-195	-3.56	-3.40	-3.48		Xudao3
	RM305-210	-0.41	-0.06	-0.23	0.00826 **	Longjing26
	RM305-215	-3.54	-3.40	-3.47		Si4230
	RM305-370	-1.69	-0.17	-0.93		Yue1
	RM3589-100	-0.40	-0.17	-0.28		Yue1
	RM3589-110	-3.19	-3.02	-3.10		Longjing26
	RM3589-115	-1.40	-1.89	-1.65		Jindao12
	RM3589-140	-2.32	-2.17	-2.25	0.00000016 ****	Huizao
	RM3589-210	-0.20	-0.39	-0.30		Yue103
	RM3589-220	-2.26	-2.46	-2.36		Yue78
	RM3589-240	-3.77	-3.73	-3.75		Nannongjing3786
	RM3589-85	-3.48	-3.70	-3.59		Cbao

	RM264-150	-6.50	-6.74	-6.62		Nannongjing2R
	RM264-155	-3.39	-4.20	-3.79		Shashani
	RM264-165	-3.60	-4.45	-4.02		Longjing26
	RM264-170	-2.00	-2.81	-2.40	0.000372 ***	Si4049
	RM264-180	-1.02	-1.75	-1.39		24248
	RM264-185	-2.32	-2.38	-2.35		Cbao
	RM264-190	-4.63	-5.97	-5.30		Nannongjing3786
	RM247-135	-1.02	-1.53	-1.27		Nannongjing3786
	RM247-150	-2.13	-1.80	-1.97		Si4049
	RM247-165	-7.63	-7.02	-7.33	0.0000795 ****	Baikenuo
	RM247-170	-2.34	-2.82	-2.58		Si4161
	RM247-185	-4.67	-4.07	-4.37		Digudao
	RM512-115	-2.71	-2.17	-2.44	0.0489*	Yue1
	RM512-220	-3.92	-3.63	-3.78		Digudao
4IN	RM301-145	-3.25	-3.13	-3.19		Nipponbare
	RM301-150	-3.46	-3.43	-3.45	0.000012 ****	Yue23
	RM301-160	-1.17	-0.97	-1.07		Longjing28
	RM301-170	-4.66	-4.78	-4.72		Sujing353
	RM348-35	-1.03	-2.11	-1.57	ns	Yue68
	RM348-155	-3.28	-3.35	-3.32		Nannongjing1R
	RM122-180	-2.64	-2.97	-2.81	ns	C418
	RM122-240	-2.39	-2.18	-2.29		Longjing26
	RM305-195	-4.09	-4.42	-4.26		Zhen9424
	RM305-205	-0.19	-0.08	-0.14	0.000944 ***	Nipponbare
	RM305-215	-2.23	-2.94	-2.58		Si4259
	RM305-370	-1.36	-0.84	-1.10		C418
	RM598-180	-3.05	-3.78	-3.42		Nipponbare
	RM225-115	-1.87	-2.77	-2.32		Nannongjing4004
	RM225-135	-0.71	-0.27	-0.49		Longjing26
	RM225-145	-1.85	-2.18	-2.01	0.000246 ***	Yue68
	RM225-155	-1.60	-2.10	-1.85		Hongnong5
	RM225-175	-4.82	-4.71	-4.77		Yandao6
	RM225-250	-2.77	-2.72	-2.74		Xudao5
	RM234-125	-2.62	-3.00	-2.81		Nannongjing2R
	RM234-130	-1.93	-1.96	-1.95		Longjing28
	RM234-140	-2.51	-2.65	-2.58		Ribenqing
	RM234-150	-0.04	-0.07	-0.05	0.000001 ****	Dongzhengwuyujing21
	RM234-155	-5.18	-5.07	-5.13		Qiuguang
	RM234-180	-0.89	-1.52	-1.21		Nannongjing4004
	RM234-200	-4.11	-4.45	-4.28		Sujing353
	RM264-150	-7.26	-7.12	-7.19		Nannongjing2R
	RM264-155	-4.87	-4.86	-4.87	0.0000209 ****	Sujing353
	RM264-165	-4.84	-4.08	-4.46		Longjing28

RM264-170	-3.23	-2.79	-3.01		Hongyin1011
RM264-180	-2.96	-2.01	-2.49		Yue49
RM264-185	-2.20	-2.57	-2.38		Nipponbare
RM264-190	-5.84	-5.92	-5.88		Nannongjing3786
RM512-115	-2.06	-2.61	-2.34	ns	C418
RM512-220	-3.42	-3.36	-3.39		Heimijingdao

*P < 0.05, **P <0.01, ***P <0.001, ****P < 0.0001, ns, not significant.

Table S13. Positive and negative elite alleles, phenotypic effect value and typical carrier materials.

Traits	Locus-alleles	Phenotypic effect value		Mean	Carrier variety
		2018	2019		
PH	RM301-130	31.05	31.01	31.03	Qiyunuo10
	RM301-140	25.64	25.36	25.5	Buxienuo
	RM301-155	25.79	25.93	25.86	Xiaomandao
	RM301-180	24.09	24.64	24.36	Yue24
	RM348-135	24.9	25.5	25.2	Yue24
	RM348-145	43.28	43.6	43.44	Maoguangdao
	RM348-170	43.86	43.31	43.58	Yue4
	RM305-105	38.91	38.67	38.79	Buxienuo
	RM264-120	39.76	39.2	39.48	Heizhong
	RM264-130	32.38	31.81	32.09	Qiyunuo10
	RM264-140	31.1	32.91	32.01	Yilimang
	RM264-160	29.06	29.29	29.17	Maoguangdao
	RM264-195	28.52	27.25	27.89	Xiaomandao
	RM512-145	28.25	27.86	28.05	Buxienuo
	RM512-165	27.99	26.15	27.07	Maoguangdao
PL	RM1-105	-0.27	-0.57	-0.42	Yue6
	RM1-110	-0.34	-0.91	-0.63	Maoguangdao
	RM1-160	-0.7	-0.72	-0.71	R254
	RM1-180	-1.67	-1.65	-1.66	Qiyunuo10
	RM1-220	-2.53	-2.95	-2.74	Nannongjing1R
	RM1-85	-4.43	-4.74	-4.59	Si4081
	RM583-160	-2.04	-1.78	-1.91	Wujing15
	RM583-175	-0.09	-0.16	-0.12	Qiyunuo10
	RM583-190	-3.04	-2.7	-2.87	Sanjiang2
	RM583-200	-1.23	-0.93	-1.08	Cbao
	RM338-100	-10.66	-10.55	-10.6	Datougui
	RM338-180	-1.55	-1.36	-1.46	Yue30
	RM338-185	-0.94	-1.6	-1.27	Qiyunuo10
	RM338-190	-2.27	-2.19	-2.23	Wujing15
	RM122-150	-5.86	-5.85	-5.85	Buxienuo
	RM122-240	-3.48	-3.07	-3.27	Yue23
	RM3330-130	-2.89	-2.98	-2.94	Hongyin1009
	RM3330-140	-1.52	-1.16	-1.34	Wumangzaodao
	RM3330-145	-0.58	0.76	0.09	Yue30
	RM3330-150	-1.94	-1.33	-1.64	Qiyunuo10
	RM3330-160	-1.01	-1.56	-1.28	Yue24
	RM3330-170	-1.59	-1.34	-1.46	R254

	RM3330-185	-1.73	-1.25	-1.49	Jinghui418
	RM3330-195	-1.16	-1.54	-1.35	Shashani
	RM3330-335	-6.06	-6.77	-6.41	Taijing9
	RM5380-100	-0.26	-0.26	-0.26	Yue30
	RM5380-105	-0.55	-0.55	-0.55	Yangfujing8
	RM5380-110	-0.79	-0.79	-0.79	Yue96
	RM5380-80	-1.02	-1.02	-1.02	Zaoshirihuangdao
	RM5380-95	-0.58	-0.58	-0.58	Qiyunuo10
	RM3533-105	-1.63	-1.22	-1.42	Zaoguangtou
	RM3533-120	-1.35	-1.18	-1.27	R254
	RM3533-130	-2.78	-2.83	-2.8	Maoguangdao
	RM3533-135	-0.35	-0.72	-0.54	Nannongjing1R
	RM3533-140	-3.04	-3.08	-3.06	Si4259
	RM3533-160	-0.32	-0.18	-0.25	Yue37
	RM3533-175	-0.62	0.13	-0.25	Nongxiang26
	RM3533-95	-6.41	-6.73	-6.57	Ningjinghui292
	RM3600-100	-2.09	-3.42	-2.76	Si4259
	RM3600-110	-1.61	-1.62	-1.62	Nongxiang26
	RM3600-120	-3.57	-3.81	-3.69	Ningjinghui145
	RM3600-80	-5.17	-5.11	-5.14	Nannongjing002
	RM3600-90	-1.03	-1.98	-1.51	R254
	RM3600-95	-0.25	-0.75	-0.5	Qiyunuo10
	RM6160-150	-3.12	-4.74	-3.93	Sanbailitou
	RM6160-175	-3.74	-3.99	-3.87	Si4259
	RM6160-185	-2.72	-2.34	-2.53	Buxienuo
	RM6160-195	-1.07	-1.99	-1.53	Qiyunuo10
1IN	RM301-145	-3.13	-3.31	-3.22	Hongbaodao
	RM301-150	-6.95	-6.29	-6.62	Zacaodao
	RM301-160	-2.6	-2.48	-2.54	Shengdao808
	RM301-170	-5.84	-4.76	-5.3	Zhongjing212
	RM264-150	-0.64	-0.69	-0.67	Nannongjing2R
	RM264-155	-6.52	-5.92	-6.22	Zhongjing212
	RM264-165	-4.74	-4.45	-4.59	Zaoxiaobaidao
	RM264-170	-5.73	-5.22	-5.48	Nannongjing1R
	RM264-185	-3.96	-3.5	-3.73	Wumangzaodao
	RM264-190	-4	-4.32	-4.16	Yue14
	RM257-135	-3.05	-3.71	-3.38	Yue91
	RM257-150	-0.47	-0.7	-0.59	Yue21
2IN	RM301-130	3.44	3.74	3.59	Cungu
	RM301-140	1.47	1.36	1.42	Zhonghua3
	RM301-155	2.37	2.01	2.19	Yue76

	RM301-180	5.1	5.36	5.23	Yue23
	RM5427-145	1.32	1.19	1.26	Longjing15
	RM5427-180	9.67	9.95	9.81	Wanjingnuo
	RM5427-200	8.3	8	8.15	Yue10(DB5)
	RM348-145	1.6	1.09	1.34	Kendao12
	RM348-170	11.23	11.42	11.33	Yue4
	RM122-270	1.01	1.51	1.26	Yue12
	RM305-105	9.7	9.49	9.59	Buxienuo
	RM305-205	1	1.8	1.4	Longjing17
	RM305-270	1.65	1.46	1.56	Zhonghua3
	RM512-145	6.41	6.02	6.22	Yue12
	RM512-165	0.22	0.83	0.53	Kendao12
3IN	RM301-130	5.67	5.5	5.58	Jianongnuo2
	RM301-140	1.12	1.9	1.51	Yue1
	RM301-155	2.63	2.14	2.39	Baikenuo
	RM301-180	3.9	3.62	3.76	Yue23
	RM348-145	3.73	3.03	3.38	Longjing26
	RM348-170	9.82	9.9	9.86	Yue4
	RM122-150	3.18	3.07	3.12	Zhonghua3
	RM122-235	0.85	0.71	0.78	Longjing28
	RM122-240	2.79	2.33	2.56	Longjing26
	RM122-270	0.73	0.93	0.83	Yue11
	RM305-105	9.79	9.41	9.6	Wanjingnuo
	RM305-205	0.26	0.96	0.61	Cbao
	RM305-270	2.03	2.18	2.1	Zhonghua3
	RM3589-125	5.09	5.16	5.13	Jianongnuo2
	RM3589-200	1.13	1.14	1.13	Yue35
	RM3589-80	1.11	1.69	1.4	Zhengdao18
	RM3589-90	3.26	3.62	3.44	Hongdao35
	RM264-120	3.42	3.98	3.7	Yishixing
	RM264-130	5.15	5.11	5.13	Jianongnuo2
	RM264-140	6.29	6.65	6.47	Yingtoudao
	RM264-160	3.73	3.74	3.74	Zaijinjing
	RM264-195	4.37	4.7	4.53	Baikenuo
	RM247-125	1.29	1.3	1.29	Sujing353
	RM247-130	3.17	3.64	3.41	H18002
	RM247-145	2.87	2.42	2.65	Zhendao88
	RM247-155	1.16	1.06	1.11	Longjing26
	RM247-160	0.12	0.14	0.13	Cbao
	RM512-130	1.54	1.51	1.53	Yue3
	RM512-145	4.3	5.53	4.92	Yue11
	RM512-165	1.9	2.1	2	Longjing26

4IN	RM301-130	4.5	4.84	4.67	Hongnong5
	RM301-140	1.7	1.43	1.57	Yue16
	RM301-155	2.77	2.33	2.55	Yue68
	RM301-180	2.83	2.46	2.65	Yue23
	RM348-130	1.88	-1.68	0.1	Yue2
	RM348-145	3.1	3.06	3.08	Longjing28
	RM348-170	11.13	14.25	12.69	Yue4
	RM122-150	4.76	4.43	4.6	Aijiaoluganhuang
	RM122-160	8.65	8.12	8.39	Yue6
	RM122-235	0.93	0.78	0.86	Longjing28
	RM122-270	1.01	1.69	1.35	Yue13
	RM305-105	9.66	9.22	9.44	Yue6
	RM305-210	0.38	0.7	0.54	Longjing28
	RM305-270	2.66	2.45	2.56	Yue13
	RM598-100	2.76	2.55	2.66	C418
	RM598-160	4.73	4.13	4.43	Yue73
	RM598-165	0.64	0.59	0.61	Longjing28
	RM598-170	10.89	10.47	10.68	Yuzhenxiang
	RM598-185	0.27	0.06	0.16	Nannongjing2R
	RM225-120	8.58	8.77	8.68	Zhen6
	RM225-125	0.24	-0.07	0.09	Yue23
	RM225-190	5.73	5.09	5.41	Lianjing2
	RM225-220	2.11	2.37	2.24	Cbao
	RM225-225	0.89	1.82	1.35	Shenlenuo
	RM225-235	5.63	5.47	5.55	R254
	RM234-100	0.21	0.48	0.34	Yue23
	RM234-105	14.01	14.3	14.15	Buxienuo
	RM234-110	3.91	3.04	3.48	Huizao
	RM234-135	3.17	3.79	3.48	Baikenuo
	RM234-160	8.88	8.15	8.52	Xueliqing
	RM234-210	8.18	8.19	8.19	Maijieqing
	RM234-290	0.81	0.81	0.81	Chiguwandao
	RM264-120	12.42	12.16	12.29	Yishixing
	RM264-130	4.13	4.42	4.27	Hongnong5
	RM264-140	7.99	7.39	7.69	Yingtoudao
	RM264-160	4.71	4.23	4.47	Zaijinjing
	RM264-195	3.15	3.67	3.41	Baikenuo
	RM512-130	1.82	1.93	1.87	Yue3
	RM512-145	5.27	5.28	5.28	Yue13
	RM512-165	1.24	1.93	1.59	Hongmangzaodao

Table S14. Prediction of excellent parental combinations, favorable allele number and increment for PH, 1IN, 2IN, 3IN and 4IN after pyramiding.

Trait	Parent/Parental combinations [a]	Nr. of favorable alleles predicted	Improvement predicted (cm)
PH	Nannongjing1R	5	-28.78
	Cai1 × Huaidao9	5	-26.23
	Cai1× Nannongjing2R	5	-30.57
	Nannongjing2R × Huaidao9	4	-26.27
	Nannongjing2R × Yue14(KDDB)	5	-29.67
PL	Yue38(70)	9	4.48
	Yue96（688） × Yue37(2)	9	3.92
	Yue96（688） × Yue85(HT9)	9	4.10
	Yue37(2) × Yue85(HT9)	8	4.47
	Yue85(HT9) × Yue63(VN2002-1)	8	4.47
PL x 1IN	Yue38(70) x Heizhong	12	7.63
	Yue38(70) x Qiyunuo10	12	9.38
	Yue96（688） x Qiyunuo10	11	8.45
	Yue37(2) x Qiyunuo10	10	9.19
	Yue38(70) x Erlibie	12	7.94
1IN	Heizhong	3	3.08
	Qiyunuo10	3	2.45
	Erlibie	3	1.73
	Yilimang	3	1.60
	Yue119（N02）	3	1.58
2IN	Hongyin1010	6	-1.18
	Hongyin1012	6	-1.29
	Si4364 × Yangmiaozhong	6	-1.02
	Si4364 × Aijiaoluganhuang	6	-2.52
	Aijiaoluganhuang × Yangmiaozhong	6	-2.53
3IN	Si4049 × Yue1（BT1）	8	-2.03
	Si4049 × Yue3(2490)	8	-1.84
	Wanjingnuo × Si4049	8	-0.98
	Si4161 × Wanjingnuo	8	-1.74
	Si4161 × Yue1（BT1）	8	-1.92
	Si4259 × Nipponbare	9	-2.29

4IN			
	Si4259 × Heimijingdao	9	-2.39
	Nannongjing2R × Heimijingdao	7	-2.63
	Nannongjing2R × Yandao6	7	-3.09
	Heimijingdao × Yandao6	7	-2.58
2IN × 3IN × 4IN	Aijiaoluganhuang × Si4161 × Nannongjing2R	11	-9.97
	Aijiaoluganhuang × Si4049 × Yandao6	12	-9.8
	Yue1（BT1）× Si4259 × Aijiaoluganhuang	11	-7.64
	Si4364 × Nannongjing2R × Si4161	11	-7.39
	Si4364 × Si4049 × Yandao6	12	-7.22

We use minus "−" for the traits of "PH", "2N", "3IN", and "4IN" to improve the plant height by reducing the height of the plant.

[a] Cultivars without the corresponding combination for cross, means this cultivar possess all of the favorable alleles at all their loci combined into a single cultivar.

Table S15. Favorable alleles carried by the superior parents for PH with its component traits and corresponding phenotypic effect.

Trait	Super parent	Locus-allele (Corresponding phenotypic effect value)			
PH	Nannongjing1R	RM301-150(-24.05)	RM348-155(-22.62)	RM305-210(-51.42)	RM264-170(-21.81)
		RM512-220(-24.00)			
	Cai1	RM301-150(-24.05)	RM348-155(-22.62)	RM305-210(-51.42)	RM512-220(-24.00)
	Huaidao9	RM301-145(-14.99)	RM305-215(-37.11)	RM264-185(-13.70)	
	Nannongjing2R	RM301-150(-24.05)	RM305-205(-41.39)	RM264-150(-33.02)	RM512-220(-24.00)
	Yue14(KDDB)	RM348-130(-33.19)	RM264-190(-24.24)		
PL	Yue38(70)	RM1-100(4.70)	RM583-180(3.24)	RM338-175(5.39)	RM122-235(1.71)
		RM3330-120(3.45)	RM5380-115(8.58)	RM3533-190(5.54)	RM3600-125(2.50)
		RM6160-160(5.17)			
	Yue96（688）	RM1-360(2.70)	RM583-180(3.24)	RM338-135(2.95)	RM122-235(1.71)
		RM3330-135(7.44)	RM3533-125(1.76)	RM3600-170(3.40)	RM6160-160(5.17)
	Yue37(2)	RM583-180(3.24)	RM338-175(5.39)	RM122-235(1.71)	RM3330-120(3.45)
		RM5380-115(8.58)	RM3600-125(2.50)	RM6160-160(5.17)	
	Yue85(HT9)	RM583-180(3.24)	RM338-135(2.95)	RM122-235(1.71)	RM3330-135(7.44)
		RM5380-115(8.58)	RM3533-125(1.76)	RM3600-170(3.40)	RM6160-160(5.17)
	Yue63(VN2002-1)	RM583-180(3.24)	RM338-175(5.39)	RM122-235(1.71)	RM3330-110(3.44)
		RM3533-220(9.44)	RM3600-125(2.50)		
1IN	Heizhong	RM301-155(4.89)	RM264-120(10.42)	RM258-130(3.15)	
	Qiyunuo10	RM301-13(5.89)	RM264-130(5.67)	RM258-130(3.15)	
	Erlibie	RM301-155(4.89)	RM264-195(5.26)	RM258-140(0.25)	
	Yilimang	RM301-155(4.89)	RM264-140(4.46)	RM258-140(0.25)	
	Yue119（N02）	RM301-155(4.89)	RM264-180(1.42)	RM258-130(3.15)	
2IN	Hongyin1010	RM301-150(-1.37)	RM5427-115(-1.42)	RM348-155(-1.22)	RM122-235(-0.27)
		RM305-210(-1.44)	RM512-220(-1.36)		
	Hongyin1012	RM301-150(-1.37)	RM5427-140(-2.05)	RM348-155(-1.22)	RM122-235(-0.27)
		RM305-210(-1.44)	RM512-220(-1.36)		
	Si4364	RM301-150(-1.37)	RM5427-135(-0.68)	RM348-155(-1.22)	RM122-240(-0.42)
		RM512-220(-1.36)			
	Yangmiaozhong	RM301-160(-2.28)	RM5427-120(-0.65)	RM348-135(-0.46)	RM122-235(-0.27)
		RM305-210(-1.44)			
	Aijiaoluganhuang	RM5427-175(-1.20)	RM348-130(-7.48)	RM122-150(-3.45)	RM305-370(-2.47)
		RM512-115(-5.55)			
	Wanjingnuo	RM122-180(-1.41)	RM3589-100(-0.28)	RM247-135(-1.27)	
	Yue1（BT1）	RM122-180(-1.41)	RM305-370(-0.93)	RM3589-100(-0.28)	RM247-135(-1.27)
		RM512-115(-2.44)			

3IN	Si4049	RM301-150(-3.72)	RM348-155(-3.42)	RM305-215(-3.47)	RM3589-100(-0.28)
		RM264-170(-2.40)	RM247-150(-1.97)	RM512-220(-3.78)	
	Si4161	RM301-150(-3.72)	RM348-155(-3.42)	RM305-215(-3.47)	RM3589-100(-0.28)
		RM264-185(-2.35)	RM247-170(-2.58)	RM512-220(-3.78)	
	Yue3(2490)	RM122-180(-1.41)	RM305-370(-0.93)	RM3589-100(-0.28)	RM247-135(-1.27)
4IN	Si4259	RM301-150(-3.45)	RM348-155(-3.32)	RM122-240(-2.29)	RM305-215(-2.58)
		RM225-155(-1.85)	RM234-130(-1.95)	RM264-185(-2.38)	RM512-220(-3.39)
	Nipponbare	RM301-145(-3.19)	RM305-205(-0.14)	RM598-180(-3.42)	RM225-135(-0.49)
		RM234-130(-1.95)	RM264-185(-2.38)		
	Heimijingdao	RM301-150(-3.45)	RM305-205(-0.14)	RM598-180(-3.42)	RM225-145(-2.01)
		RM234-150(-0.05)	RM264-185(-2.38)	RM512-220(-3.39)	
	NannongingR2	RM301-150(-3.45)	RM305-205(-0.14)	RM225-155(-1.85)	RM234-125(-2.81)
		RM264-150(-7.19)	RM512-220(-3.39)		
	Yandao6	RM301-145(-3.19)	RM305-215(-2.58)	RM598-180(-3.42)	RM225-175(-4.77)
		RM234-130(-1.95)	RM264-185(-2.38)		
